# Biochar-based organic substrates enhance tomato growth by promoting specific microbial communities in rooftop farming

**DOI:** 10.1186/s40793-025-00744-z

**Published:** 2025-07-01

**Authors:** Miguel de Celis, Laura L. de Sosa, Giuseppe Picca, Noelia González-Polo, Cristina Gómez Ruano, Luciano Beneduce, Claudio Zaccone, Marco Panettieri

**Affiliations:** 1 Department of Soil, Plant and Environmental Quality, Institute of Agricultural Sciences (ICA-CSIC), C/ de Serrano 115b, Madrid, 28006 Spain; 2 Department of Biotechnology, University of Verona, Strada Le Grazie 15, Verona, 37134 Italy; 3 Department of the Science of Agriculture, Food, Natural Resources and Engineering (DAFNE), University of Foggia, Via Napoli, Foggia, 25 -71122 Italy

## Abstract

**Background:**

Urban agriculture represents an opportunity to alleviate some of the issues related with the increased urbanization and global human population. Productive rooftops offer a promising solution that combines food production and recycling of organic waste, while providing green spaces without consuming urban soils. In addition, harnessing natural resources, including plant-associated microbiomes, is proposed as an effective approach to sustainably improve farm productivity and food quality. However, little attention has been given to the importance of selecting the appropriate organic substrate to enhance plant-microbe interactions and rooftop farming in urban areas. Here, we study the influence of different organic substrates on tomato, and its associated microbial community, in an open-air rooftop farming setup. Specifically, we evaluate combinations of peat with a high quantity (50% of volume) of compost derived from different feedstocks (seaweed biomass or coffee byproducts), and how biochar-blending alters these compositions.

**Results:**

We found that substrate properties were mainly defined by the compost feedstocks. Biochar blending had a minor influence on substrate composition, although it enhanced tomato yield. Overall, alternative substrates differed from peat mainly by showing higher phosphorus content, pH, and electric conductivity. Similarly, compost feedstock had a greater impact on microbial communities than biochar blending. Even though alternative substrates presented higher bacterial diversity than peat, the taxonomic composition was similar across alternative substrates, reflecting the functional redundancy of the bacterial communities. In addition, we identified specific microbes associated with each compost feedstock. The abundance of bacteria and fungi associated with composted seaweed biomass was positively associated with tomato yield. Among them, bacterial genera like *Acidibacter*, *Altererythrobacter*, *Amaricoccus*, *Luteitalea*, *Microvirga*, *Pedomicrobium* or *Pseudorhodoplanes* stood out presenting strong correlations with tomato yield.

**Conclusions:**

The studied substrates influenced tomato growth and yield directly via their chemical and physical properties and by influencing microbial community composition. Thus, our results reveal the importance of using the appropriate organic substrate for enhancing the effectiveness of rooftop agriculture while increasing microbial diversity in urban unexploited areas.

**Supplementary Information:**

The online version contains supplementary material available at 10.1186/s40793-025-00744-z.

## Background

The increase in the world population is accompanied by a sustained growth of the urban population, particularly relevant in emerging economies and developing countries due to rural-urban migration [1]. Urbanization results in a range of undesired consequences, including environmental pollution and soil degradation [2]. Urban agriculture can help alleviate urban challenges associated with climate change, soil degradation and food security, while aligning with the United Nations Sustainable Development Goals (SDGs). Particularly, SDGs 2 (zero hunger), 3 (good health and wellbeing), 6 (clean water and sanitation), 13 (climate action), and 15 (life on land), which are interconnected and promote sustainable farming practices. In this context, productive rooftops have emerged as a promising solution for “zero acreage farming”, a form of agriculture that occupies unused urban spaces and does not require traditional farmlands [3]. Rooftop systems can provide various services including urban temperature regulation, air purification, organic waste recycling, esthetic satisfaction, and, most notably, food supply [4, 5]. Harnessing natural resources in a circular biobased economy context, like plant-associated microbes and producing organic substrates from residues, is proposed as an effective approach to sustainably improve agriculture, while also delivering positive environmental and social benefits [6]. Tomato is one of the most widely consumed vegetables worldwide and is relatively easy to grow [7] making it an excellent model crop. Thus, this crop allows for the study of the benefits provided by rooftop agriculture. It also enables the exploration of potential sustainable practices, such as microbiome engineering and the use of alternative organic substrates, to enhance plant growth and yield in these systems.

Peat is a main component in various substrates used for soilless horticultural cultivation [8]. However, it is considered a non-renewable resource, with a high environmental impact associated with its extraction [3]. Using alternative organic materials can help reduce the peat usage while increasing the availability of nutrients and water for plants and maintaining high microbiological activity [9–11]. Biowastes are widely used as constituents of organic horticultural substrates because of their potential for boosting fertility and contributing to climate change mitigation [12]. Among these, organic residues from the geotextile or agronomic industries, and biochar stand out [13–15]. Biochar could further enhance the efficiency of the composting process, modifying the surface chemistry of end products and the plant interaction with nutrients and soluble organic matter [16–18]. However, microbial communities dwelling alternative organic substrates have received less attention, although both aspects are important for better understanding the interactions between substrates and plants [19]. In addition, the microbial ecosystem services that arise from natural interactions between agricultural soils and surrounding environments, such as forests or meadows, are largely absent in urban agriculture [20]. This lack of exchange further highlights the role of organic substrates and the careful evaluation of soil microbiomes become crucial for sustaining soil fertility, nutrient cycling, and plant health in these constrained urban settings.

Microbial communities constitute a highly complex and dynamic ecosystem [21]. Plant-associated microbes are fundamental to plant growth with crucial roles in plant health and protection from diseases [22, 23]. Many studies have addressed the positive effects of composts and biochar on soil microbiomes, including their ability to promote microbial activities, enhance microbial biomass, increase diversity of specific functional groups, or improve biogeochemical processes [19, 24–27]. These effects are driven by the different physical and chemical properties among substrates. Differences in pH among compost and biochar-compost substrates can affect microbial communities, promoting bacterial diversity and, to a lesser extent, fungal community composition [28, 29]. Organic matter quantity and quality, along with its carbon and nutrient content, is related to microbial activity and fungal abundance [30]. However, the appropriate selection of organic substrates to enhance plant-microbe interactions and rooftop farming in urban areas have received little attention.

In this study, we aimed to evaluate the quality of alternative organic substrates derived from compost made from different compost feedstock materials (seaweed biomass or coffee byproducts), and how biochar-blending alters these compositions (Supplementary Figure S1). We focused on how these alternative substrates enhanced tomato production directly, and through the associated microbial communities. We measured the substrate properties and bacterial/fungal composition at the beginning of the experiment and after the tomato harvesting. By using tomato yield as a comprehensive metric, we summarized the indirect and direct effects of substrate production on plant growth and its associated microbial diversity, crucial for sustainable agriculture. This single-harvest experiment highlights the critical importance of selecting the appropriate organic substrate for enhancing the effectiveness of rooftop agriculture.

## Methods

### Substrate composition and experimental design

The urban rooftop used for this study was situated in Madrid at the ICA-CSIC building (40°30′00″N, 3°40′24″W; https://madreenroof.csic.es/*).* Six composting mixtures were prepared, as outlined in Table 1. The bio-wastes used included spent coffee grounds (SCG), coffee silverskin (CS), and seaweed (SW), prepared with and without biochar (BC) blending. All bio-wastes were co-composted with green waste (GW) from garden pruning. SCG was collected from the coffee vending machines at the Institute of Agricultural Sciences (ICA) - CSIC (Madrid, Spain), CS was provided by Café Candelas SA (Lugo, Spain), SW was harvested from the Atlantic beach of “Playa de la Costilla” in Rota (Cádiz, Spain), and GW was supplied by a local pruning company in Brunete (Madrid, Spain). BC was obtained from Carbón Vivo SL (Barcelona, Spain) and produced by pyrolysis of Aleppo pine pruning waste at 650–750 °C for 3.5 h.

**Table 1:** Volumetric proportions of materials used for the six composting mixtures (bulk densities represented in Supplementary table S4) and their corresponding ratios

Composting mixture	Spent Coffee Grounds (SCG)	Coffee Silverskin (CS)	Seaweed (SW)	Green Waste (GW)	Biochar (BC)	Mixture ratio (Feedstock: GW: BC)
SCG-BC	1	0	0	1	1	1/1/1
SCG	1	0	0	1	0	1/1/0
CS-BC	0	1	0	1	1	1/1/1
CS	0	2^a^	0	1	0	2/1
SW-BC	0	0	1	1	1	1/1/1
SW	0	0	1	1	0	1/1/0

^a^ Compost CS started with a ratio 1:1 (v: v) of CS and GW; a second dose of CS was added halfway through the process (15 days) to overcome the compaction of CS during composting, reaching a final CS: GW ratio of 2/1 (v/v). Doses are equal to approximately 38 L of the material.

SCG: Spent Coffee Grounds, CS: Coffee Silverskin; SW: Seaweed; GW: Green Waste; BC: Biochar; -BC: biochar blended substrates.

Mixtures derived from coffee by-products (SCG and CS) were composted at ICA-CSIC in 200 L composters equipped with a passive aeration system (HOTBIN composting, Northampton, UK), as previously described by Picca et al. [31]. The mixtures were composted for 30 days and left in open air for another 30 to 90 days to reach maturity. Seaweed-based mixtures underwent composting in windrow piles at the “Compost Ecológico” facility in Rota, Spain, following the method described by Madejón et al. [32]. After composting, all mixtures were ground using a garden shredder (Viking GE 105) to obtain particles smaller than 10–20 mm. Substrates were prepared by combining the mixtures with a commercial peat (Jiffy GO PP7, Jiffygroup) at a 50% (v/v) ratio.

The substrates were placed into 70 L plastic pots (44 cm tall and 49 cm wide diameter) using two different architectures according to Grard et al. [3].: (1) a bottom layer of biochar (19 cm), topped with a compost/peat mixture (19 cm), and (2) a bottom layer of biochar (5 cm), mimicking clay balls, followed by a single layer of biochar-blended compost/peat (33 cm) (Supplementary Figure S1). Control pots were prepared using the first configuration using the commercial peat media. Substrates were sampled at the beginning and end of the crop cycle for physical, chemical, and biological characterization [33].

### Chemical and physical analysis of substrates

Physical and chemical properties were analyzed on a 100 g fresh aliquot collected from each pot replicate (*n* = 5) and dried at 40 °C on a drying and sterilization oven (J.P. Selecta, Spain) for three days before characterization. The pH and electrical conductivity (EC) were measured in water extracts (1:10 w/v) using a CRISON micropH 2001 and a CRISON microCM 2201 (Crison Instruments SA, Barcelona, Spain), respectively. For EC, extracts were filtered through syringe PTFE filters at 0.45 μm pore size.

Total Carbon (C) and Nitrogen (N) were analyzed by dry combustion and gas chromatography using a ThermoFlash 2000 NC Soil Analyzer (Thermo Fisher Scientific, Waltham, MA, USA).

Total potassium (K) and phosphorous (P) contents were assessed by digesting the samples with hydrochloric acid (HCl) and nitric acid (HNO_3_) in a Mars One microwave (Allenium, Barcelona, Spain). The resulting extract was then analyzed with an ICP-OES Varian 720-ES (Agilent Technologies, Inc., Santa Clara, CA, USA) at the IRNAS-CSIC analytical service (Seville, Spain).

### Agronomic performance of substrates

The substrates agronomic performance was tested by growing a local variety (from the Community of Madrid) of tomato (*Solanum lycopersicum* L., cv. Moruno de Aranjuez) from May to September 2021. Tomato plantlets, provided by the Instituto Madrileño de Investigación y Desarrollo Rural, Agrario y Alimentario (IMIDRA), were transplanted when they had developed three sets of true leaves, reaching approximately 15 cm in height, consistent with both IMIDRA’s protocols and widely accepted horticultural guidelines. Plant height was measured weekly. Tomatoes were harvested at full ripeness to assess the total yield for each treatment.

### Microbial community assessment

The biological characteristics, diversity and composition of bacterial and fungal communities, were determined by amplicon sequencing at the “López-Neyra” Institute of Parasitology and Biomedicine analytical service (IPBLN-CSIC, Granada, Spain). Briefly, 10 g samples were initially stored at -20 °C, then freeze-dried with a LyoQuest-55 (Telstar, Terrassa, Spain) and finally ground with a Mixer Mill MM400 (Retsch GmbH, Haan, Germany). The DNA was extracted using the DNeasy^®^ 96 PowerSoil^®^ Pro Kit (QIAGEN N.V., Hilden, Germany) from 125 mg of substrate sample. Extracts were sent to the IPBLN-CSIC where DNA quality and quantity was checked using NanoDrop 2000 (Thermo Fisher Scientific, USA) and Qubit Fluorometer (Thermo Fisher Scientific, USA), respectively. Library preparation and amplicon sequencing were carried out using different primer sets for each microbial group studied. The bacterial 16 S ribosomal rRNA gene (V3-V4 region) was amplified using 16 S ProV3V4 forward (CCTACGGGNBGCASCAG) and 16 S ProV3V4 (GACTACNVGGGTATCTAATCC) primer set and the nuclear ribosomal internal transcriber spacer (ITS) with primers FITS7 (GTGARTCATCGAATCTTTG) and ITS4 (TCCGCTTATTGATATGC). Libraries were subsequently sequenced on Illumina^®^ MiSeq instrument (Illumina, USA) following manufacturer’s instructions.

Bioinformatic analyses were conducted to obtain high-quality chimera-free bacterial and fungal sequences in the R environment [34]. Sequence analysis was performed with *dada2* v1.26.0 R package [35] which allows for the identification of amplicon sequence variants (ASVs), distinguishing true biological variation from sequencing errors and PCR artefacts [35]. This enhances the identification of unique microbial taxa and the quantification of their abundances. Primers were removed using *cutadapt* [36] and *Biostrings* v2.66.0 R package [37] to assess their orientation within the reads, low-quality ends were deleted, and no mismatches were allowed when merging paired reads. Once chimaeras were removed, taxonomy was assigned to the 16 S reads using the SILVA v138.1 database [38] and the ITS reads using the UNITE v9.0 database [39]. A total of 4,069,336 good quality bacterial sequences, averaging 58,133 ± 9598 per sample, and 6,056,854 fungal sequences, averaging 86,526 ± 12,660 per sample, were obtained. Archaeal reads accounted for 0.025 ± 0.0463% abundance, so from now on we will refer to the prokaryotic community as bacterial community.

### Diversity and statistical analysis

Diversity indices were calculated after rarefaction. Briefly, each sample was subsampled to 36,997 bacterial and 47,909 fungal reads (Supplementary Figure S2), repeated this process 100 times and averaged resulting alpha and beta diversity metrics [40]. Alpha diversity was calculated using Hill-based diversity index, which allow to incorporate the importance given to the relative abundance of each ASV [41] using the *hillR* v0.5.2 R package [42]. This importance is determined by the diversity order, and allow us to calculate the species richness, the exponential Shannon index (Hill-Shannon) and reciprocal Simpson index (Hill-Simpson) [43]. Differences in community composition across samples (β-diversity) were evaluated with the *vegan* v2.6-6.1 R package [44] by calculating Bray-Curtis dissimilarity matrices, and performing non-metric multidimensional scaling (NMDS) to compress dimensionality into two dimensional plots.

Community structure was addressed calculating co-occurrence networks using a probabilistic model [45]. As we aimed to calculate the structure of the metacommunity across treatments, keeping in mind that the main microbial inoculum comes from the same commercial peat, we first collapsed the ASV table to the genus level and filtered out those present in less than one-third of the samples. 498 of 1243 bacterial genera (accounting for 96.67 ± 1.73% of reads) and 174 of 762 fungal genera (accounting for 98.01 ± 2.09% of reads) were used in further analysis. Co-occurrence networks consisted of a total of 429/17,196 and 158/2824 bacterial and fungal nodes/edges, respectively. The function *cluster_waltrap* from the *igraph* v 2.0.3 R package [46] was used to determine each node’s module membership. In addition, the method developed by Ortiz-Álvarez et al. [47] was applied to calculate the individual networks of each sample and calculated the proportion of each assigned module in each individual network (that is, module completeness).

Permutational multivariate analysis of variance (PERMANOVA) and variance partitioning analysis were used to assess the effect of several variables on substrates (Euclidean distances) and microbial communities composition (Bray-Curtis dissimilarities) using the *vegan* R package [44]. Random forest analysis was run using the *randomForest* v.4.7–1.1 R package [48] to identify the relative importance of the different chemical substrate and microbial properties predicting tomato yield production. These models make use of bootstrap and out-of-bag (OOB) samples to estimate errors and explained variance (R²) [49]. Predictor importance is additionally assessed by the increase in error when a variable is excluded [48]. To address whether the effects of compost feedstock material and biochar application method on substrate properties and microbial communities translates into changes in tomato yields, structural equation modelling (SEM) was used. First, we hypothesized a conceptual causal model focusing on the bacterial and fungal taxa positively correlated with tomato yield. We accounted for the variation in multiple substrate initial properties driving microbial communities, such as organic matter content, nutrient concentrations (N, P and K) and substrate properties (pH and EC). We created variables accounting for the bacterial and fungal modules positively correlated with tomato yield. Information about our a priori model is provided in the Supplementary Figure S3 and Supplementary Table S3. We calculated a linear model for each endogenous variable in the mode and used the *psem*, within the *piecewiseSEM* v2.3.0 R package [50] to unite all the structural equations into a single structural equation model. The goodness of fit was assessed with the Fisher’s C-test, to test if the model is a causal scenario consistent with the data (*p*-value > 0.05). Code for diversity and statistical analyses is available at https://github.com/Migueldc1/Roof.

## Results

### Influence of compost feedstock and Biochar blending on substrate properties

**Fig. 1:**
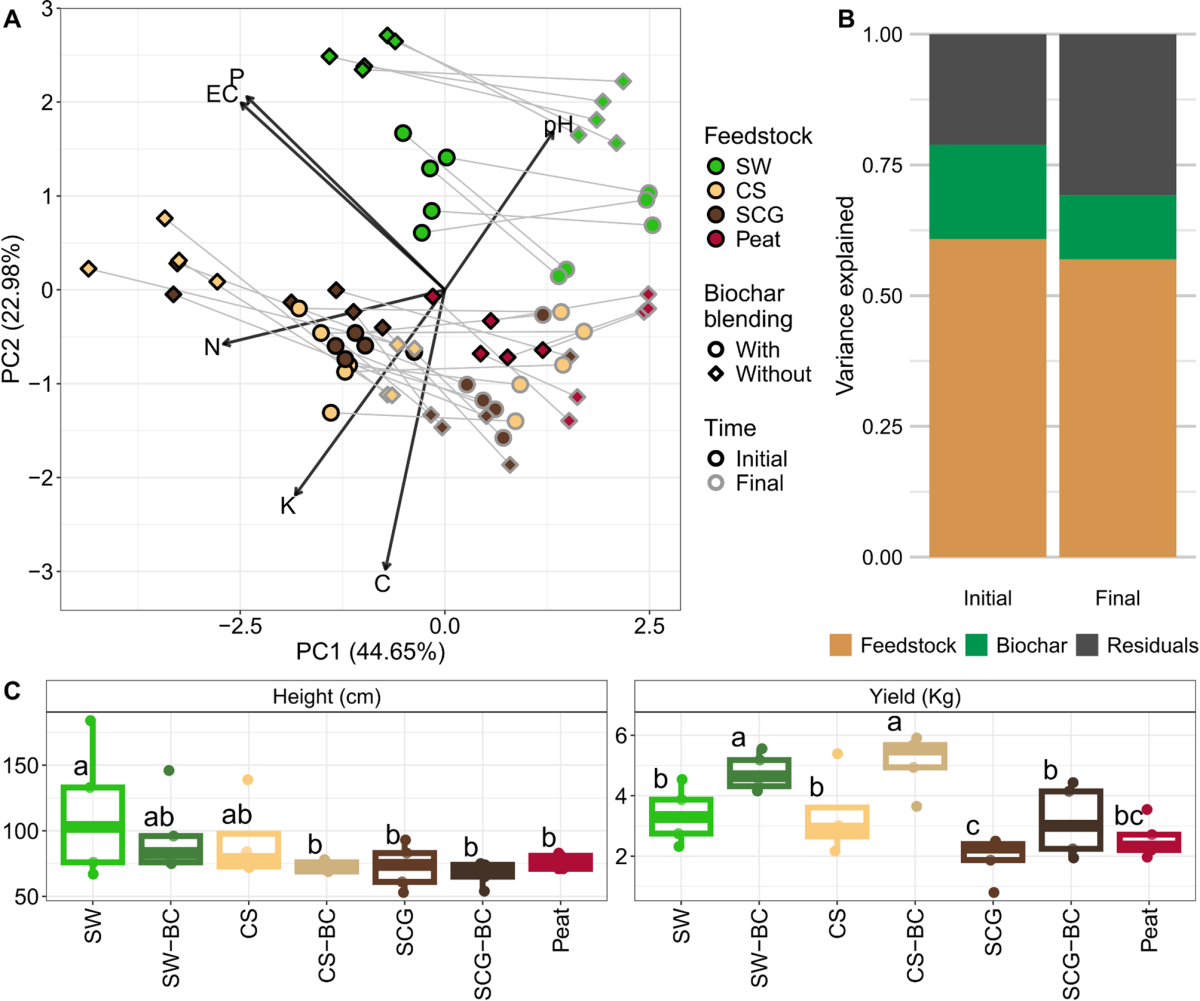
Chemical properties of alternative organic substrates and tomato plant growth. **(A)** Principal Component Analysis (PCA) based on substrate properties addressing differences among compost feedstock materials (denoted by colors) and biochar application (denoted by shapes). Circles and diamonds represent whether biochar was blended with compost or added at the bottom of the pot, respectively. Each sample was measured before (black border) and after (gray border) tomato cultivation, both sampling points are joined with a grey line. Arrows indicate each individual measurement of substrate properties, and their length indicates the contribution of each variable. **(B)** Variance Partitioning Analysis describing the percentage of variation in substrate composition explained by compost feedstock material and biochar application method, as well as their shared effect if any. Variation not explained (residuals) is also shown. **(C)** Influence of alternative organic substrates on tomato growth measured as plant height (cm) and yield (kg tomato per plant). ANOVA test and LSD (Least Square Difference) test were conducted (a-c indicate significance groups). SW – Seaweed, CS – Coffee Silverskin, SCG – Spent Coffee Grounds, BC – Biochar was blended during composting

The analyzed substrates, from different compost feedstock materials and biochar-compost blending (-BC), presented a different composition (Fig. 1A**B**). Most of the variance was explained by the compost feedstock material, while the biochar application explained a lower proportion of the total variance (Fig. 1B). The alternative substrates differed from peat mainly by presenting higher P content, pH and EC (Fig. 1A, Supplementary Figure S4). The composts derived from coffee by-products–Spent Coffee Grounds (SCG) and coffee silverskin (CS) – had, in general, high organic C and N content (Fig. 1A, Supplementary Figure S4). The most different substrate from peat was the composted seaweed (SW) waste, which also presented the highest differences between compost and biochar-compost substrate (Fig. 1A). SW-BC substrate presented higher C content and lower N content than SW substrate.

These applied substrates also altered tomato growth, producing significant differences in plant height and harvest yield (Fig. 1C). Even though we observed a high variability within treatments, peat-based control and SCG induced lower yields (Fig. 1C). We did not find a generalized correlation between plant height and yield (Supplementary Figure S5A). Tomato yield was higher in biochar-compost blended substrates. During tomato growth, the substrates composition changed because of nutrient leaching during watering and uptake by plant (Fig. 1A**B**, Supplementary Figure S5B). Specifically, N, P, K and EC decreased after harvest, whereas pH and the C/N ratio increased (Supplementary Figure S5B). Organic C content remained constant across all treatments. These changes seemingly made substrate composition more similar among treatments (Fig. 1A), which is also denoted by the lower distances among substrates at the end of the experiment (Wilcoxon test, *p* = 0.02).

### Effect of organic substrates on microbial communities

**Fig. 2:**
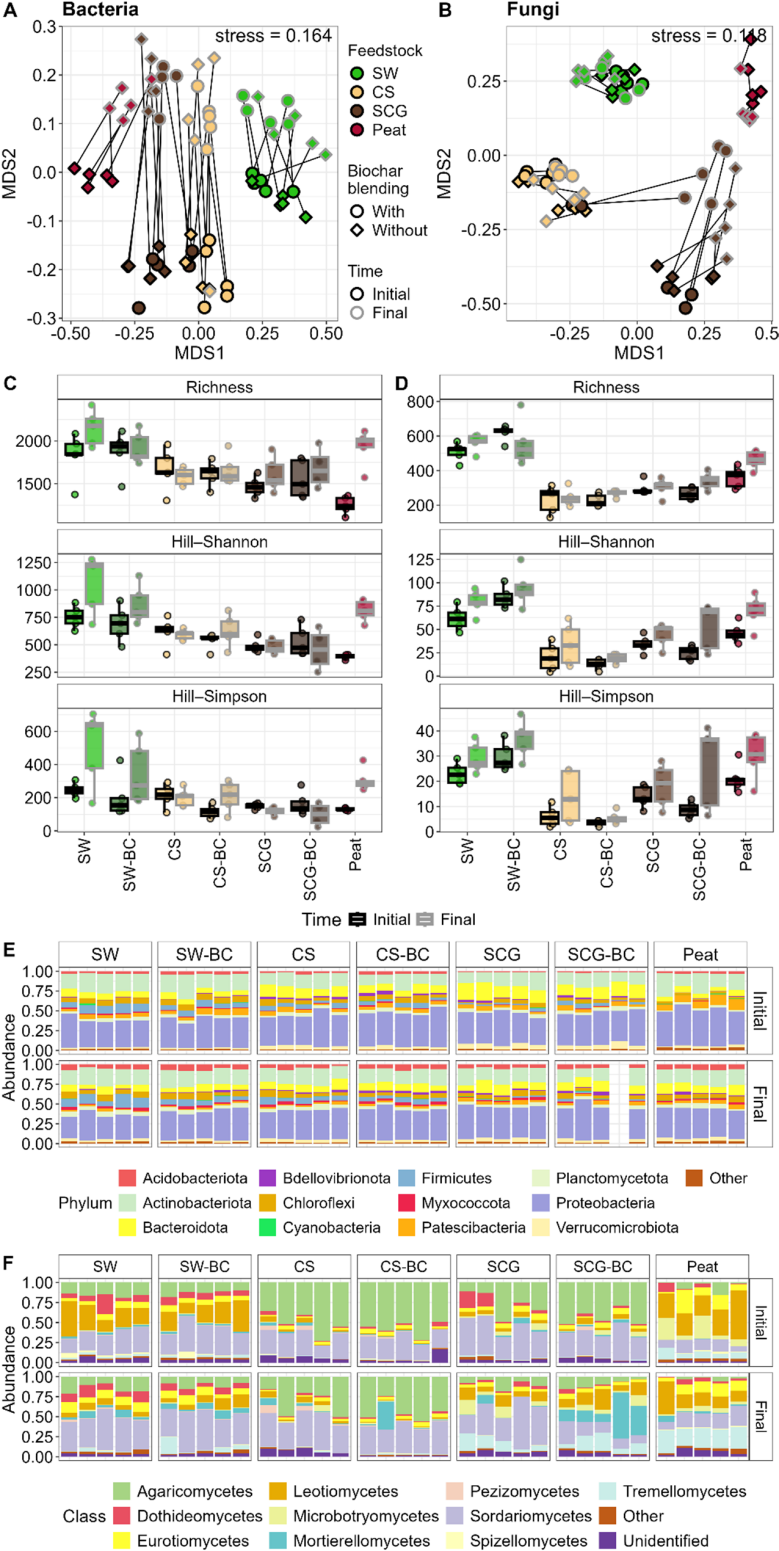
Combined effect of compost-biochar on shaping bacterial and fungal community composition and diversity. Non-metric multidimensional scaling (NMDS) plots, based on Bray-Curtis dissimilarity matrices representing the structure of **(A)** bacterial and **(B)** fungal communities before and after tomato cultivation. Black lines join sampling points (before and after tomato cultivation). The effects of compost feedstock material, biochar application method and tomato cultivation on communities were examined by permutational multivariate analysis of variance (PERMANOVA; Supplementary Table S1). Richness Hill-Shannon and Hill-Simpson diversity indices as measurement of alpha diversity of **(C)** bacterial and **(D)** fungal communities. Taxonomic composition of **(E)** prokaryotic communities at the phylum level and **(F)** fungi communities at the class level. “Other” includes minor prokaryotic phylum (relative abundance lower than 2.5%) and fungal class (lower than 5%), and “Unidentified taxonomically unassigned taxa. SW – Seaweed, CS – Coffee Silverskin, SCG – Spent Coffee Grounds, BC – Biochar was blended during composting

The composition of bacterial and fungal communities differed among substrates, with compost feedstock material explaining the highest variance, especially in the fungal communities (Fig. 2**AB**, Supplementary Table S1). Biochar application also exerted significant influences on microbial communities but was less relevant than compost feedstock material (Supplementary Table S1). Bacterial community composition at the phylum level was similar among growing substrates, being dominated by *Proteobacteria* and *Actinobacteriota* (Fig. 2**EF**). Noticeably, peat samples presented a higher proportion of *Patescibacteria* while substrates based on coffee derived feedstocks, SCG(-BC) and CS(-BC), presented a higher proportion of *Bacteroidota*. Fungal communities, on the other hand, presented a different composition at the class level depending on the compost feedstock material (Supplementary Table S5B). SCG and CS, even with differences, presented a higher proportion of *Agaricomycetes* and *Sordariomycetes*, the latter also dominating in SW samples. *Leotiomycetes* and *Eurotiomycetes* were especially relevant in SW and peat samples. Compared to the commercially available peat-based substrate, compost-based substrates increased bacterial diversity by up to 48% in SW-based substrates, which were the only ones to also show an increase in fungal diversity, achieving around a 56% rise, while biochar had minimal impact on these changes (Fig. 2**CD**).

Microbial composition changed during the growth of tomato, especially bacterial communities (Supplementary Table S1). We observed a generalized increase in the relative abundance of *Acidobacteria* and *Chloroflexi* bacterial phyla (Fig. 2E). Fungal community succession was characterized by a generalized decrease in the abundance of *Leotiomycetes* and increases of *Sordariomycetes*, *Mortierellomycetes* and *Tremellomycetes* (Fig. 2F). Interestingly, the bacterial and fungal diversity increased after tomato growth, especially in peat-based samples (Fig. 2**CD**, Supplementary Figure S6).

### Compost feedstock material shapes microbial community structure

**Fig. 3:**
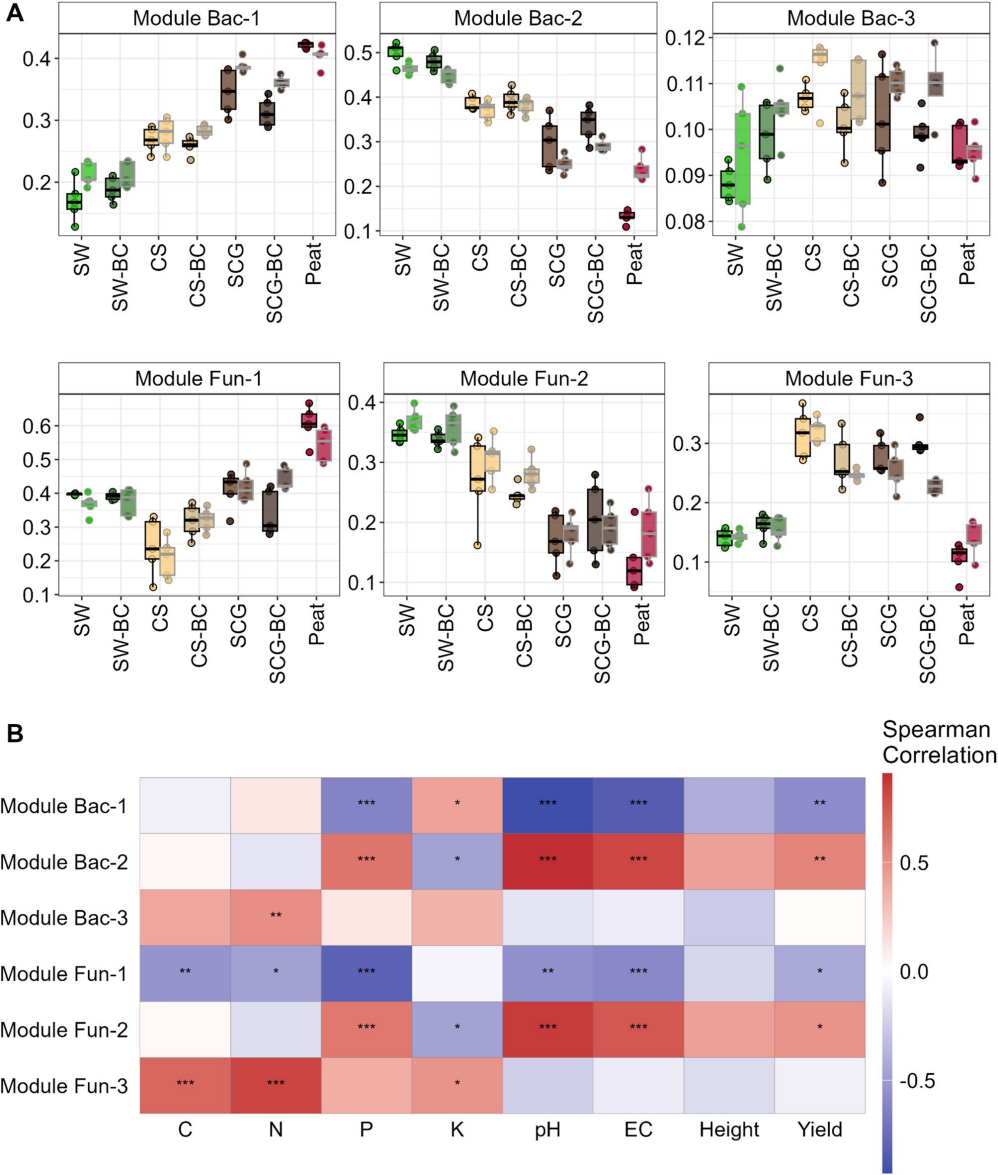
Microbial sub-communities across different compost feedstocks and biochar application, and their relationship with substrate properties. **(A)** Module completeness, the proportion of nodes belonging to a given module present in each individual sample. **(B)** Spearman’s rank correlation coefficient between bacterial and fungal module completeness and substrate and tomato properties. Asterisks denote the significance of each pairwise correlation (false discovery rate adjusted, *p < 0.05, **p < 0.01, ***p < 0.001)

Both bacterial and fungal communities were divided into three network modules, resembling sub-communities, with varying proportions among growing substrates (Fig. 3A). Bacterial and fungal sub-communities followed similar patterns in module completeness, the proportion of nodes belonging to a given module, with module/sub-community 1 being dominant in peat samples, module 2 dominating in SW and CS samples and module 3 outstanding in SCG and CS samples (Fig. 3A). Interestingly, this pattern remains unaltered even after tomato growth (Fig. 3A). In addition, bacterial-fungal bipartite network reveals trends compatible with parallel community succession driven by interkingdom interactions (Supplementary Figure S7).

Both bacterial and fungal module 2 correlated positively and significantly with tomato yield and substrate properties such as P content, EC and pH (Fig. 3B). The taxonomic composition of these modules differed from the complete communities (Supplementary Figure S8). Bacterial module/sub-community 2 presented a higher proportion of *Acidobacteriota* and *Firmicutes*, especially in SW and CS samples, highlighting the increased proportion of *Verrucomicrobiota* and *Bacteroidota* in SCG and CS samples and peat. In general, *Dothideomycetes* and *Mortierellomycetes* fungal classes presented higher proportion compared to the complete community, as well as *Sordariomycetes*, with the only exception of SGC samples.

### Organic substrates drive tomato yield through physical and chemical properties, and microbial community composition

**Fig. 4:**
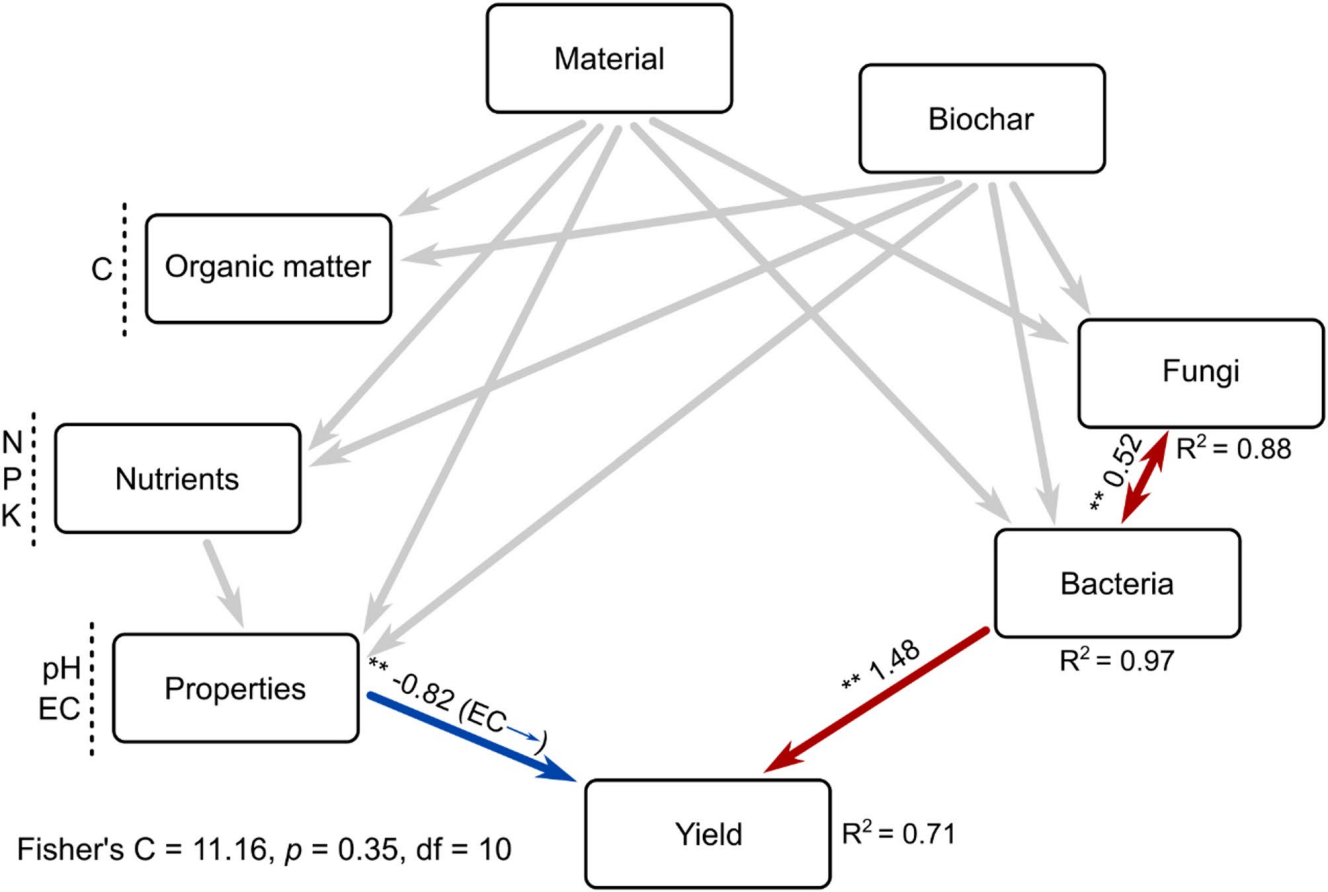
Direct and indirect effects of compost feedstock material and biochar application method on tomato yield. Structural Equation Model (SEM) revealing the direct and indirect effects of compost feedstock material and biochar application method on substrate initial properties, microbial communities, and tomato yield. ‘Bacteria’ and ‘Fungi’ represent the proportion of bacterial and fungal communities potentially associated with plants (Fig. 3), respectively. ‘Nutrients’, and ‘Properties’ are the joint effects of multiple variables, which were grouped in the same box for graphical simplicity. ‘Material’ refers to the compost feedstock material used in each treatment, while ‘Biochar’ indicates the biochar application method (either at the bottom of each pot or blended during composting). Parentheses indicate which specific observed variable within a box has a direct effect on a response variable. Arrows represent significant associations, and adjacent numbers indicate the standardized coefficient of significative paths (*p < 0.05 and **p < 0.01). Red and blue arrows represent positive and negative paths, respectively, among properties and tomato yields. Grey arrows represent significant associations not involving tomato yield (provided in Supplementary Table S2 for graphical simplicity). R2 represents the proportion of variance of a response variable explained by all predictors. Fisher’s C statistic refers to the overall goodness of fitness, with a high *p* value indicating good fitness of the model to the data. C, organic carbon content (%); N, total nitrogen content (%); P, total phosphorous content (ppm); K, total potassium content (ppm); EC, electric conductivity (mS/m). The rationale behind our a priori model is provided in Supplementary Figure S3 and Supplementary Table S3

Structural Equation Model revealed that tomato yield was directly influenced by specific bacterial communities and substrate initial properties (EC) even after accounting for fungal communities, organic and nutrient content and the unmeasured effect of the substrate composition–the combination of compost feedstock materials and biochar application – (Fig. 4). We found a strong correlation between bacterial and fungal communities, corresponding to both modules 2, even after accounting for the putative influence of substrate composition (Fig. 4, Supplementary Figure S9A). We did not find any direct effect of substrate chemical composition and properties on bacterial and fungal communities in SEM analysis (Fig. 4), while P, K, EC and pH correlated significantly with the proportion of bacterial and fungal communities (Supplementary Figure S9A). Organic feedstocks influenced nutrient and C substrate composition, directly affecting its properties. Substrate properties and chemical composition were also correlated, revealing that both are important for tomato yield. EC and pH, together with bacterial module 2 proportion, were the major predictors of tomato yield in a random forest analysis (Supplementary Figure S9B).

**Fig. 5:**
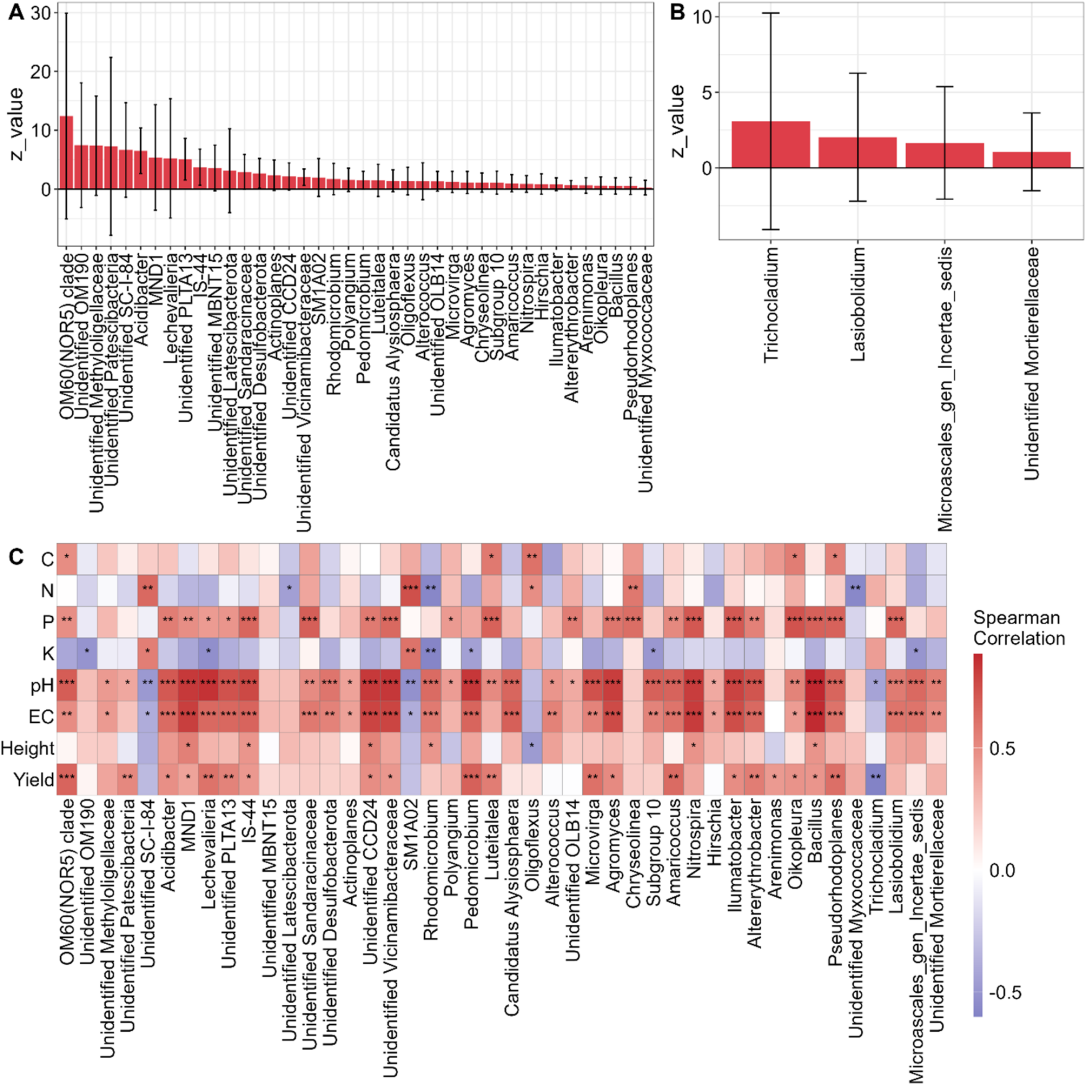
Bacterial and fungal genera associated tomato yield. **(A)** Bacterial and **(B)** fungal general belong to Module-2 with increased abundance after tomato harvest. **(C)** Correlations between bacteria and fungi abundance with substrate properties and tomato yield and growth

We identified bacterial and fungal genera with increased proportion after tomato growth (Fig. 5). Among them, bacterial genera like *Acidibacter*,* Altererythrobacter*,* Amaricoccus*,* Luteitalea*,* Microvirga*, *Pedomicrobium* or *Pseudorhodoplanes* stood out because of the strong correlations with tomato yield. Concerning fungal communities, we did not find any genera positively correlated with tomato yield, but they could still be influencing plant performance through indirect effects through substrate properties or bacterial communities.

## Discussion

The effects of alternative substrate properties on microbial communities, plant production, and productivity in rooftop agriculture remain largely unexplored, despite their potential to alleviate urbanization-related concerns while providing alternative and sustainable food production. Substrate properties were mainly defined by the compost feedstock material while biochar-compost blending enhanced the quality of the corresponding substrate, resulting in higher tomato yields. Concerning microbial communities, the biochar application method was less relevant, as most of microbial community variance was explained by composted feedstock. In addition, we identified specific communities associated with each composted material, being those associated with composted seaweed biomass, and to a lesser extent with coffee silverskin, positively and significantly correlated with tomato yield. Overall, compost feedstock material and biochar application method influenced tomato production by determining substrate properties and the microbial diversity associated. Thus, obtained results reveal the importance of using the appropriate organic substrate for enhancing the effectiveness of rooftop agriculture.

### Biochar-compost blending enhances the quality of substrates

The present study revealed that biochar blending during composting had little effect on substrate properties, compared to compost feedstock materials. Coffee derived substrates (SCG and CS) are of great interest for agriculture, seeking an improvement in plant growth, seed germination, or nutrient content of the vegetables, because of their high C and nutrient content [51, 52]. Among them, CS substrates presented higher salinity (revealed by the higher EC) and P than SCG, probably due to cations and/or nutrients being washed out during coffee brewing [53]. Seaweed-based substrates were defined by lower C and N content and a higher pH. Previous studies highlight the release of quickly biodegradable C and potential N losses, the latter accompanied by pH increases, during seaweed composting [54, 55]. Seaweed compost has been reported to be rich in sodium (Na), leading to higher values of EC and pH [56]; however, seaweed bloom characteristics are very site-specific and the SW compost used for this study did not show this issue. Concerning biochar application, the main differences found were related to higher total C content, accompanied by higher C/N ratio in biochar-blended substrates, which is attributed to the high stability of biochar C [31, 57]. In turn, C/N ratios in biochar-blended substrates were more similar to peat samples. These results suggest that the organic substrates analyzed represent potential substitutes of peat to make organic substrates that reduce our dependence on finite resources and fragile ecosystems.

In general, nutrient content decreased after tomato harvest across all substrates, especially N and P, while pH and the C/N ratio increased. The degree of nutrient loss varied among substrates, highlighting the lower loss of P in peat. Despite this, total P contents were still higher for all the substrates at the end of the experiment, compared to peat, indicating no P limitation for the crop development. Even though no major differences concerning nutrient loss were found between compost and biochar-blended composts, we found higher tomato yields in compost-biochar blended substrates. Compost-biochar blending is described to generally improve substrate specific properties such as C content, pH or water retention, depending on the compost feedstock material [58–60] which could enhance tomato production. This suggests that while the compost feedstock material primarily defines compost characteristics, biochar would act as a key modifier, enhancing the overall performance of the substrate. This makes biochar-blended substrates particularly suitable for rooftop farming systems where optimizing water and nutrient management is crucial.

### Alternative organic substrates drive microbial communities

Microbial community composition responded to the differences in the properties of the organic substrates, with compost feedstock material exerting a greater impact than biochar application method. These findings align with previous research indicating that the type of organic compost feedstock used during composting plays a crucial role in shaping microbial community structures, which in turn influence plant health and productivity. The composted materials provide specific nutrients and support diverse microbial populations that interact with plant roots, often enhancing nutrient availability and promoting plant growth [61]. The observation that the biochar application method had less of an impact on microbial variance is consistent with findings suggesting that biochar influence on microbial communities can depend heavily on the composition of the organic material it is combined with, rather than biochar alone [62, 63]. Overall, organic treatments showed higher bacterial diversity compared to peat samples, especially seaweed substrates, which also presented higher fungal diversity. These substrates are usually rich in diverse organic compounds such as vitamins, proteins or carbohydrates, which could enrich microbial diversity [64]. Compost generally increases the variety of nutrients and ecological niches of peat alone, which would explain the higher bacterial diversity. Interestingly, the bacterial community composition at the phylum level remained similar to previously described peat-based substrates [65, 66]. Even though alternative substrates presented higher bacterial diversity than peat, their taxonomic composition remained similar among treatments, reflecting the functional redundancy of the bacterial communities. Fungal communities, on the other hand, exhibited more distinct compositions depending on the compost feedstock material, although richness was comparable between coffee derived substrates and peat. Fungi seemed to be more responsive to physical factors like different substrate texture across treatments [67] as well as the composition of organic matter. In this context, the presence of complex organic molecules (such as lignocellulose in coffee silverskin or spent coffee grounds substrates) and complex polysaccharides (such as alginate or laminarin in seaweed substrates) likely favors the development of specialized fungal communities [53, 68–71] which may explain the observed differences in composition.

The parallel structure of bacterial and fungal communities also suggests that each compost feedstock material promotes the development of specialized communities. We identified three main sub-communities, each associated with seaweed, coffee derived or peat substrates. Among these, the seaweed-related sub-community correlated significantly and positively with tomato yield. Interestingly, while the bacterial composition of this sub-community was somewhat similar to that of the broader microbial community, fungal composition differed markedly. Notably, this sub-community was enriched with fungi from the classes *Mortierellomycetes*, *Dothideomycetes* and *Sordariomycetes*. Fungi, potentially with plant growth promoting activities, belonging to these classes have been found enriched in previous studies assessing the fungal communities associated with tomato [72–75]. In addition, several genera belonging to the *Sordariomycetes* class are among the beneficial endophytic fungi inoculated to enhance horticultural crops, including tomato [76]. Organic substrates play a crucial role in introducing and supporting microbial communities that plants can harness to improve health and growth [77]. Therefore, selecting the appropriate organic substrate could be helpful for the selection of beneficial microbes that enhance tomato production.

### Substrate properties and microbial communities modulate tomato yield

We identified bacterial and fungal sub-communities associated with tomato yield, which accounted for the higher proportion of the community in seaweed and coffee silverskin substrates. These sub-communities were correlated with P and K content, as well as with EC and pH, which are key factors influencing microbial growth and activity [78–80]. This is also supported by random forest analysis, which reveals that pH and EC, together with bacterial and fungal sub-communities, were the main factors driving tomato yield. Among these, EC was the only substrate factor that directly affected tomato yield in the SEM analysis, and its negative influence may explain the higher yield observed in biochar-blended substrates, which had lower EC values compared to their counterparts. The negative effect of too high initial EC on tomato yield is a well-known phenomenon, particularly in substrates where salinity might interfere with nutrient uptake and plant water balance [81, 82]. Additionally, biochar can act as a buffer by improving water retention and reducing salinity [83]. However, CS samples were among the higher tomato producers, even though their initial EC values were the highest. This could be related to the higher nutrient content, or the higher porosity of CS-substrates, compared with SW-substrates, the latter exerting positive effects in the water cycle and reducing the compaction of the substrate [51] which could promote a better EC reduction during plant growth and resulted in higher tomato production.

The SEM analysis revealed that the fungal sub-community indirectly influenced tomato yield by affecting the bacterial sub-community. In this sense, we did not find any fungal genera with increased abundance after harvest that directly correlated with tomato yield, although they may still play roles in shaping the tomato microbiome through the hydrolysis of various polymers abundant in the substrates studied [77]. Within the bacterial sub-community, however, several genera showed higher abundances after harvest and presented positive and significant correlations with tomato yield and other substrate parameters such as P, K, EC and pH. These genera could be directly enhancing plant growth, like the *Bacillus* genus, which is well-known and widely used as a microbial inoculant to enhance the development of different crops [84, 85]. However, future research is needed to identify the specific functional contribution of *Acidibacter*, *Altererythrobacter*, *Amaricoccus*, *Luteitalea*, *Microvirga*, *Pedomicrobium*, *Luteitalea*, *Microvirga*, *Amaricoccus*, *Altererythrobacter* or *Pseudorhodoplanes* that our study correlated with increased tomato yield. A characterization of the strains having the most relevant plant growth-promoting or biocontrol activities could be harnessed to improve tomato growth and yield.

### Future scope

This study highlights the potential of alternative organic substrates to improve rooftop agriculture through improvements in plant performance and microbial communities. We need to expand research in several directions to optimize substrates for urban rooftop agriculture. In particular, testing combinations of organic residues and composting strategies will help optimize substrate formulations for both plant productivity and microbial activity. However, it is still needed to consider factors like the structural load-bearing capacity of the host building or the nutrient dynamics to develop substrates suitable for long-term use. Importantly, developing substrates using locally available organic waste streams, such as those proposed in the present study, can support the principles of a circular bioeconomy, reduce environmental impact while creating value-added products tailored to specific urban contexts.

## Conclusions

In rooftop farming scenarios, where the choice of substrate must balance the needs for sustainability, productivity, architectural suitability and efficient resource use, alternative organic substrates offer a promising solution. Our results underscore that biochar, when blended with compost feedstock materials such as seaweed biomass and coffee by-products, can significantly improve the functional properties of the growing media, potentially leading to more sustainable and productive urban agriculture systems. The different compost feedstock materials assayed increased the microbial diversity compared to commercially available substrate and favored the development of specialized microbial communities with different influences on tomato yield. In particular, the seaweed-associated communities seemingly exerted direct effects on yield, providing a strong foundation for exploring specific bacterial genera with potential applications in urban agriculture. Additionally, the reduced environmental footprint associated with utilizing waste products like seaweed and coffee residues further strengthens the case for biochar-compost blends in urban agriculture. Adopting these substrates not only boosts crop yields but also aligns with broader goals of circular economy and increases biodiversity and climate resilience in urban food production.

## Electronic supplementary material

Below is the link to the electronic supplementary material.

Supplementary Material 1

## Data Availability

The datasets generated and/or analyzed during the current study are available in a GitHub repository, https://github.com/migueldc1/Roof. Raw files are available in the National Center for Biotechnology (NCBI) repository under the project code PRJNA1176355 (reviewer link https://dataview.ncbi.nlm.nih.gov/object/PRJNA1176355?reviewer=33vo6j8poa2tu8vhsncralr799).
