# Supplementary material for: Biochar-based organic substrates enhance tomato growth by promoting specific microbial communities in rooftop farming
Source: Environ Microbiome. 2025 Jul 1;20:82. doi: 10.1186/s40793-025-00744-z (PMC12220821; doi:10.1186/s40793-025-00744-z)
Supplement: Supplementary file 1 — Supplementary Material 1 [file 40793_2025_744_MOESM1_ESM.docx]

**Supplementary Material**

**
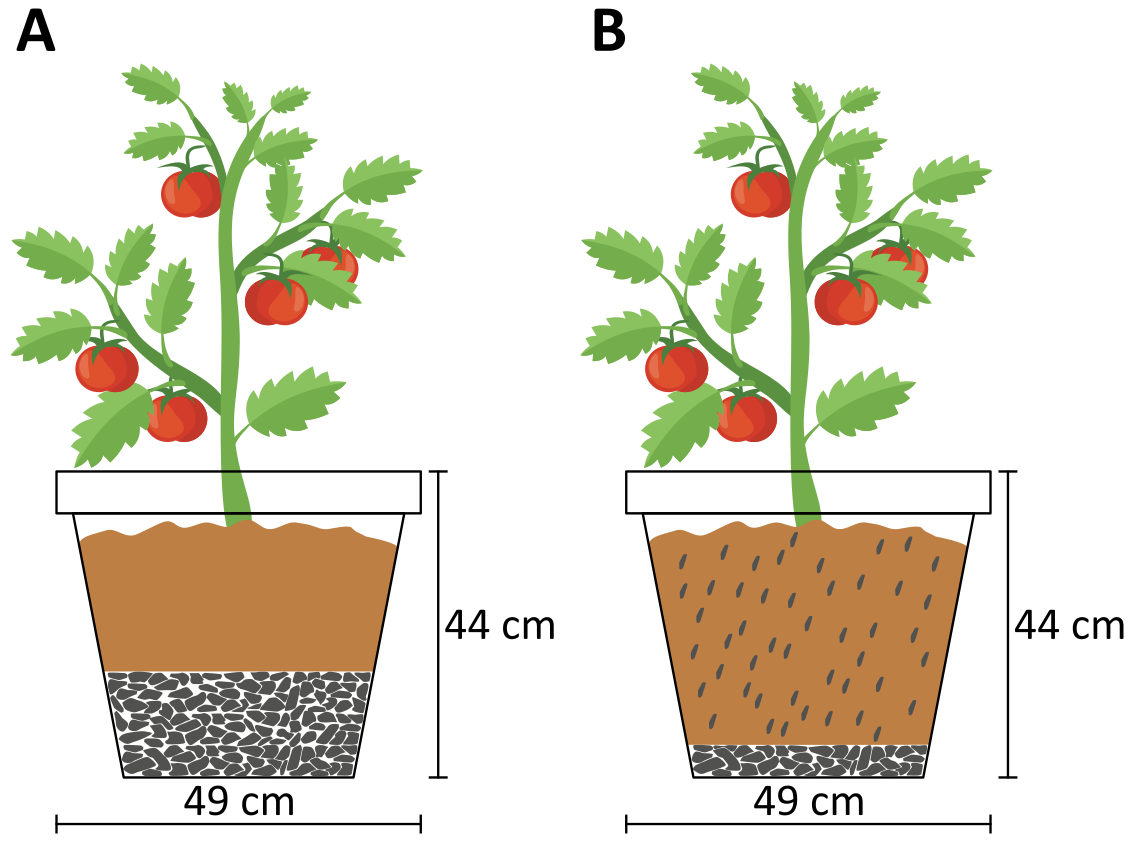
**

**Supplementary Figure S1.** Depiction of the two architectural models used in substrate preparation: A) a bottom layer of biochar (19 cm), topped with a compost/peat mixture (19 cm); B) a bottom of biochar (5 cm), mimicking clay balls, followed by a single layer of biochar-blended compost/peat mixture (33 cm).


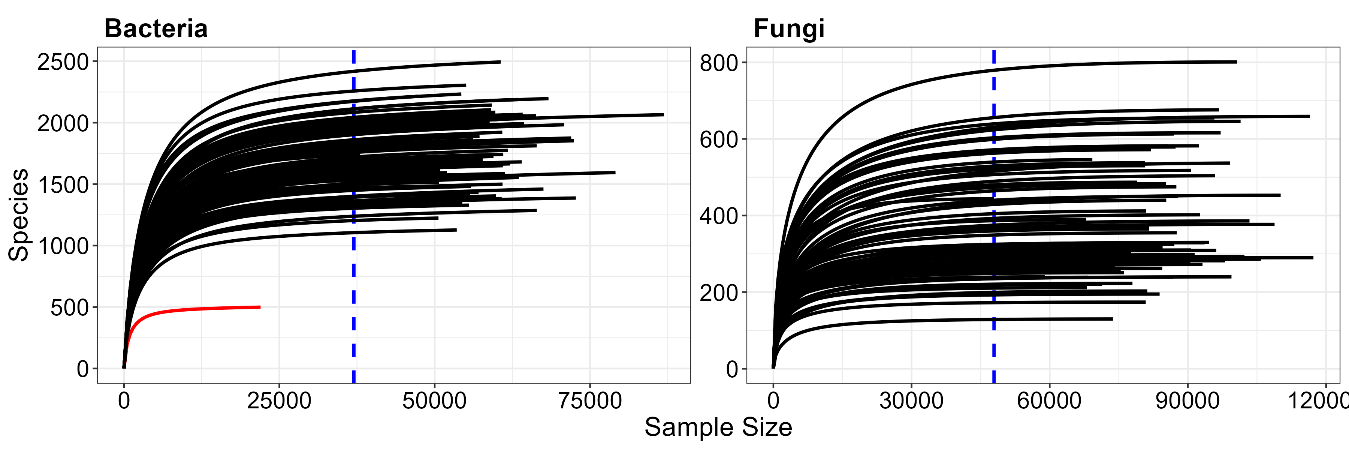


**Supplementary Figure S2.** Rarefaction curves showing the number of unique ASVs as a function of normalized library size for all samples. Red curves represent samples with small library sizes and thus, excluded from downstream analysis (missing bar in **Figure 2E**).


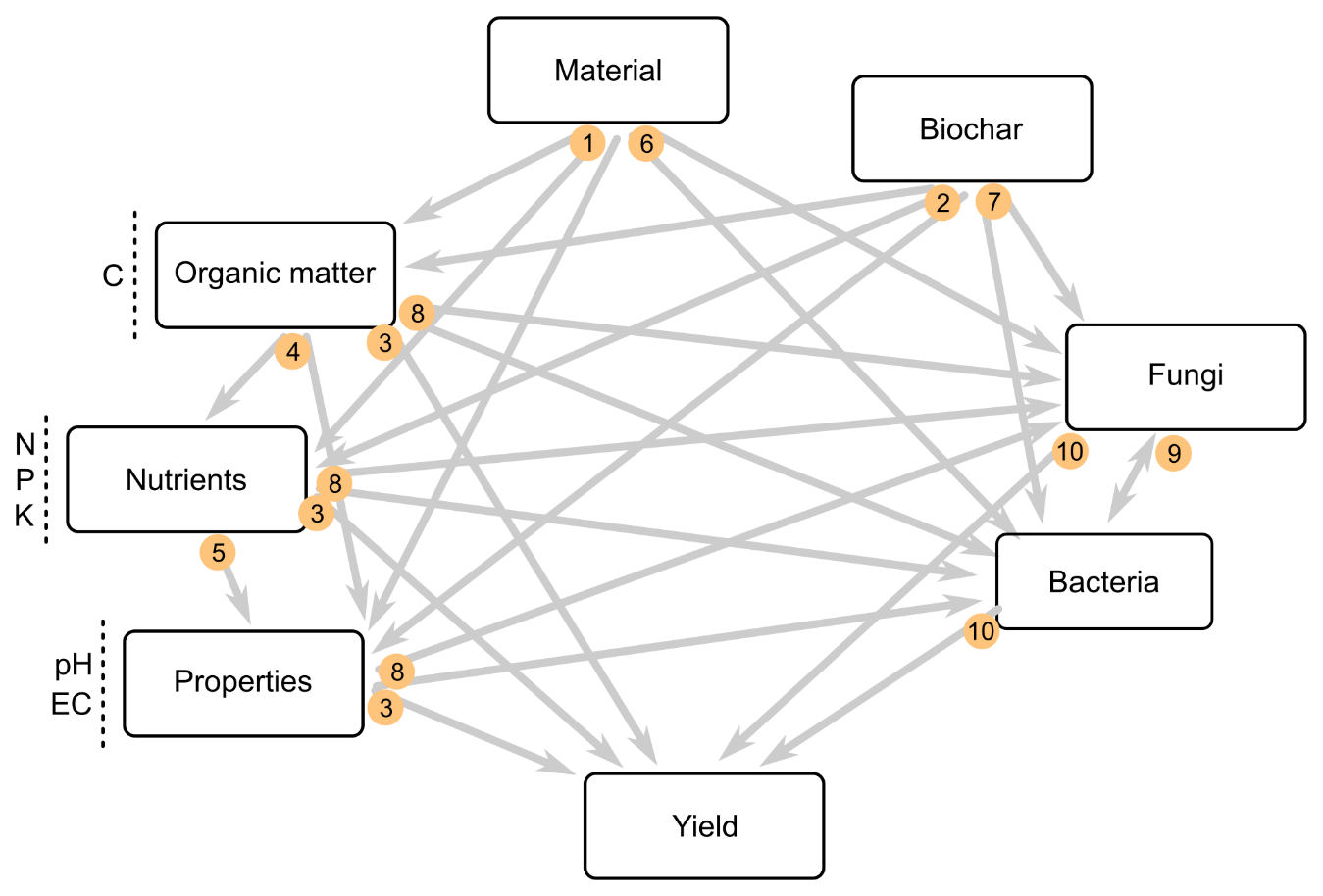


**Supplementary Figure S3.** A priori structural equation modeling (SEM) metamodel aimed to evaluate the direct and indirect effects of feedstock materials and biochar application on substrate properties, microbial communities, and tomato yield. Numbers indicate the rationale behind each association link explained in Supplementary Table S3.

**
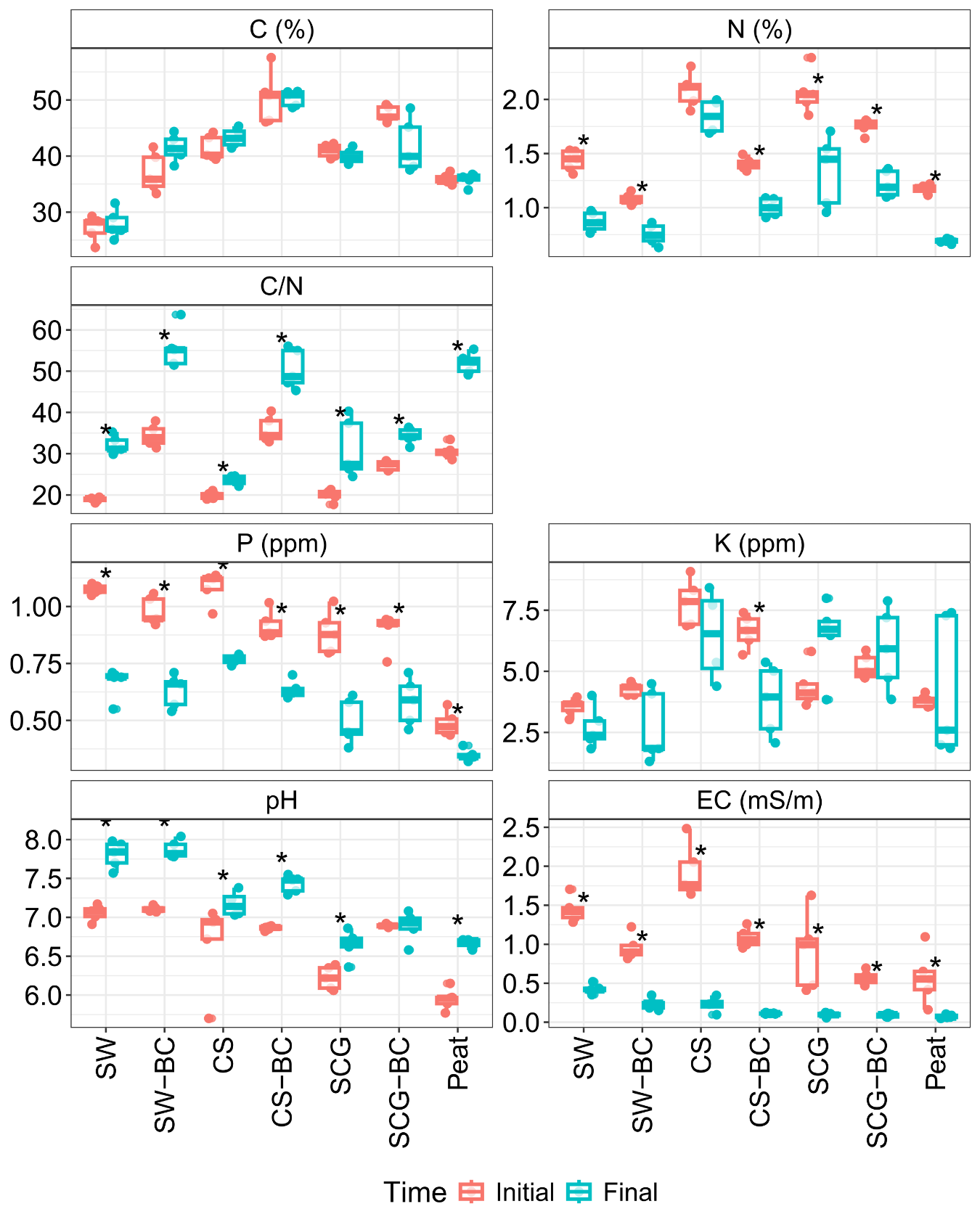
**

**Supplementary Figure S4.** Substrate composition and chemical properties after tomato cultivation. Asterisks denote the significance differences between substrate properties before and after tomato cultivation (Wilcoxon, **p* < 0.05). SW – Seaweed, CS – Coffee Silverskin, SCG – Spent Coffee Grounds, BC – Biochar was blended during composting. EC – Electric Conductivity, C/N – Carbon/Nitrogen ratio.


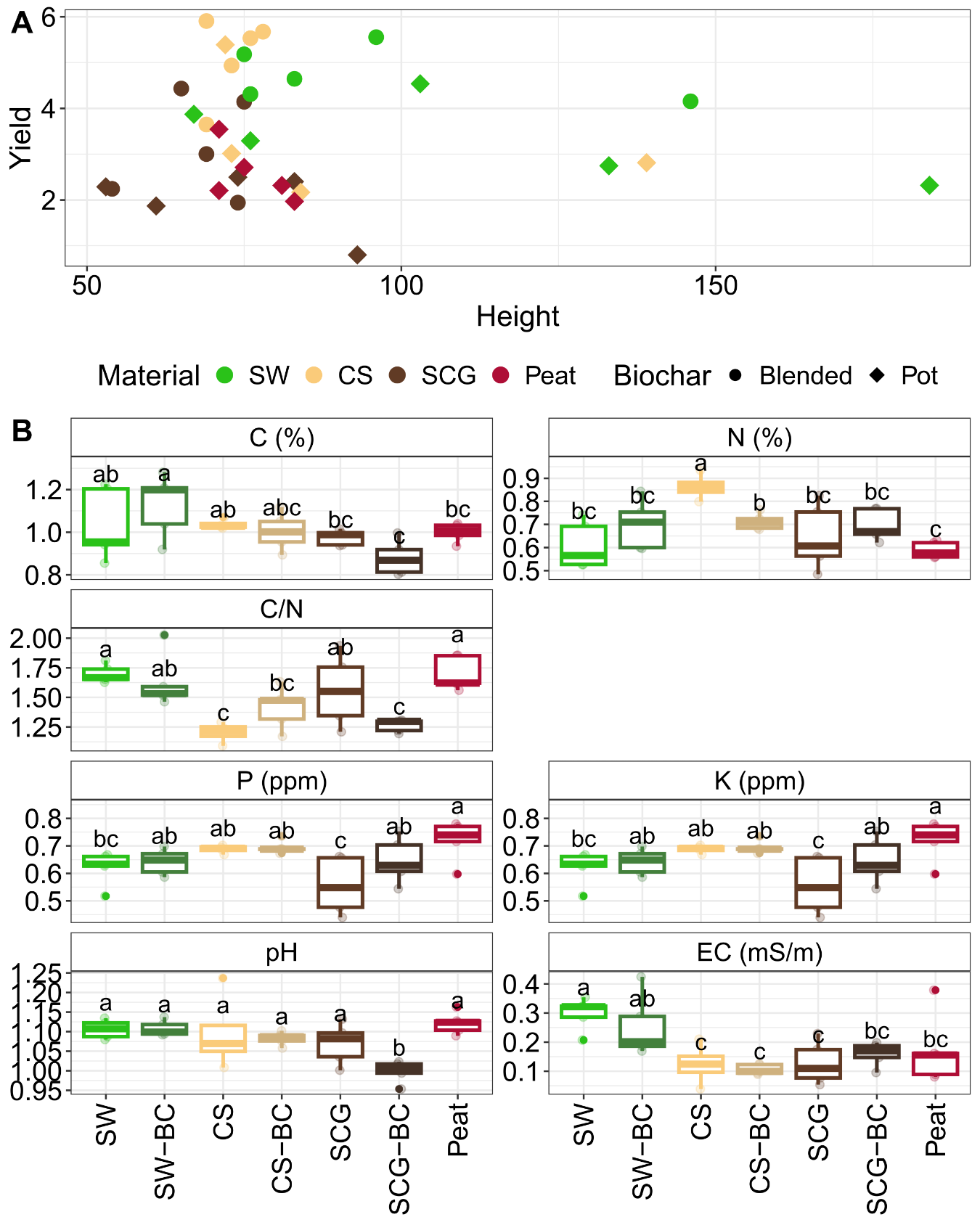


**Supplementary Figure S5.** **A)** Scatter plots showing the relationships between tomato yield (kg) and height (cm). **B)** Changes in substrate composition and chemical properties after tomato cultivation across treatments. SW – Seaweed, CS – Coffee Silverskin, SCG – Spent Coffee Grounds. ANOVA test and LSD (Least Square Difference) test were conducted (a-c indicate significance groups).


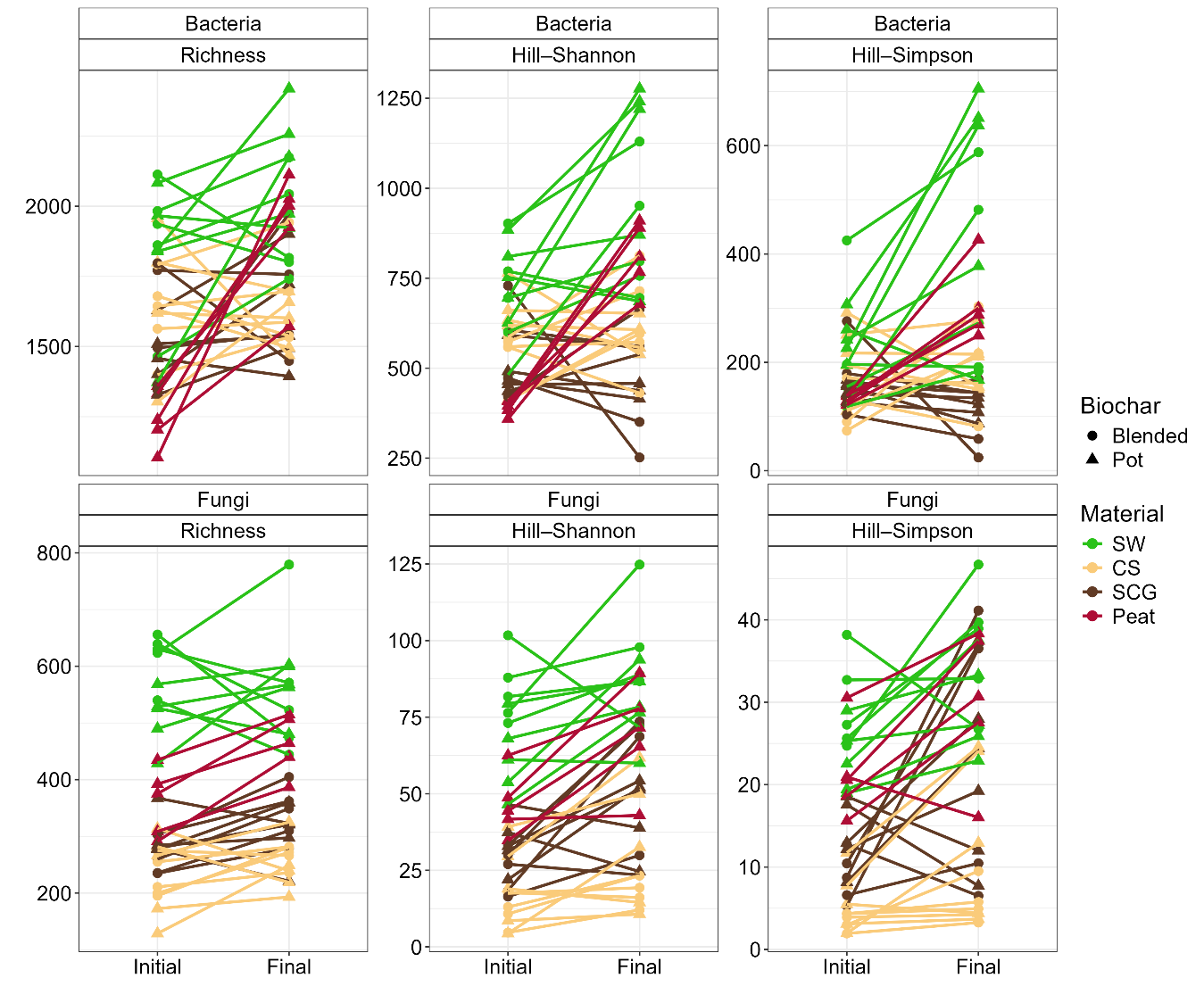


**Supplementary Figure S6.** Increasing alpha diversity after tomato cultivation is mediated by the combined effect of compost-biochar, that is, treatment. SW – Seaweed, CS – Coffee Silverskin, SCG – Spent Coffee Grounds.


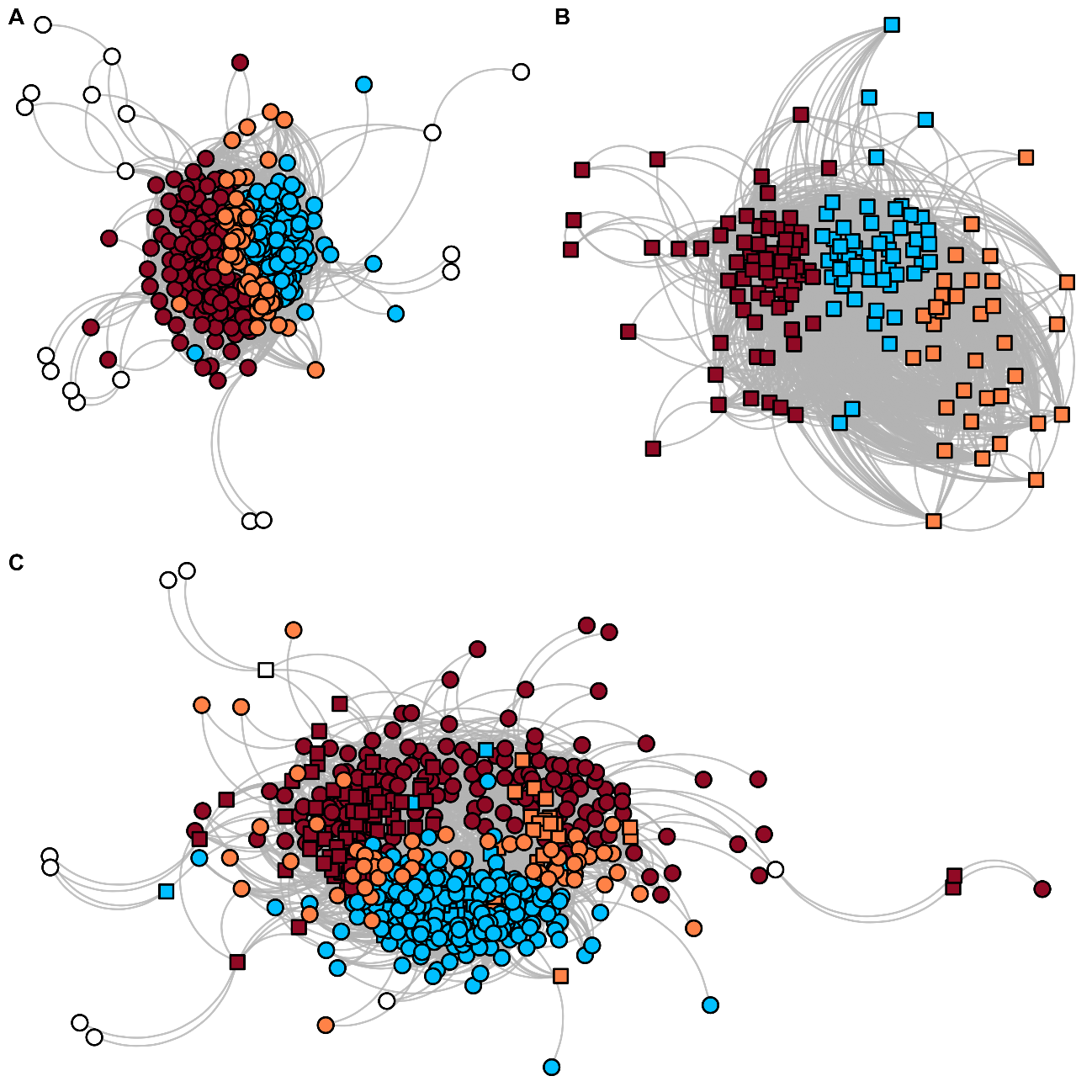


**Supplementary Figure S7.** Co-occurrence networks revealing **A)** prokaryotic and **B)** fungal sub-communities and **C)** their interaction in a bipartite network. Network nodes represent community members (aggregated at the genus level) and edges significant relationships between nodes (probability of co-occurrence higher than 0.95). Nodes are colored based on their module membership, being the prokaryotic and the fungal networks divided in three different modules (Module Bac-1: red, Module Bac-2: blue and Module Bac-3: orange; and Module Fun-1: red, Module Fun-2: blue and Module Fun-3: orange).


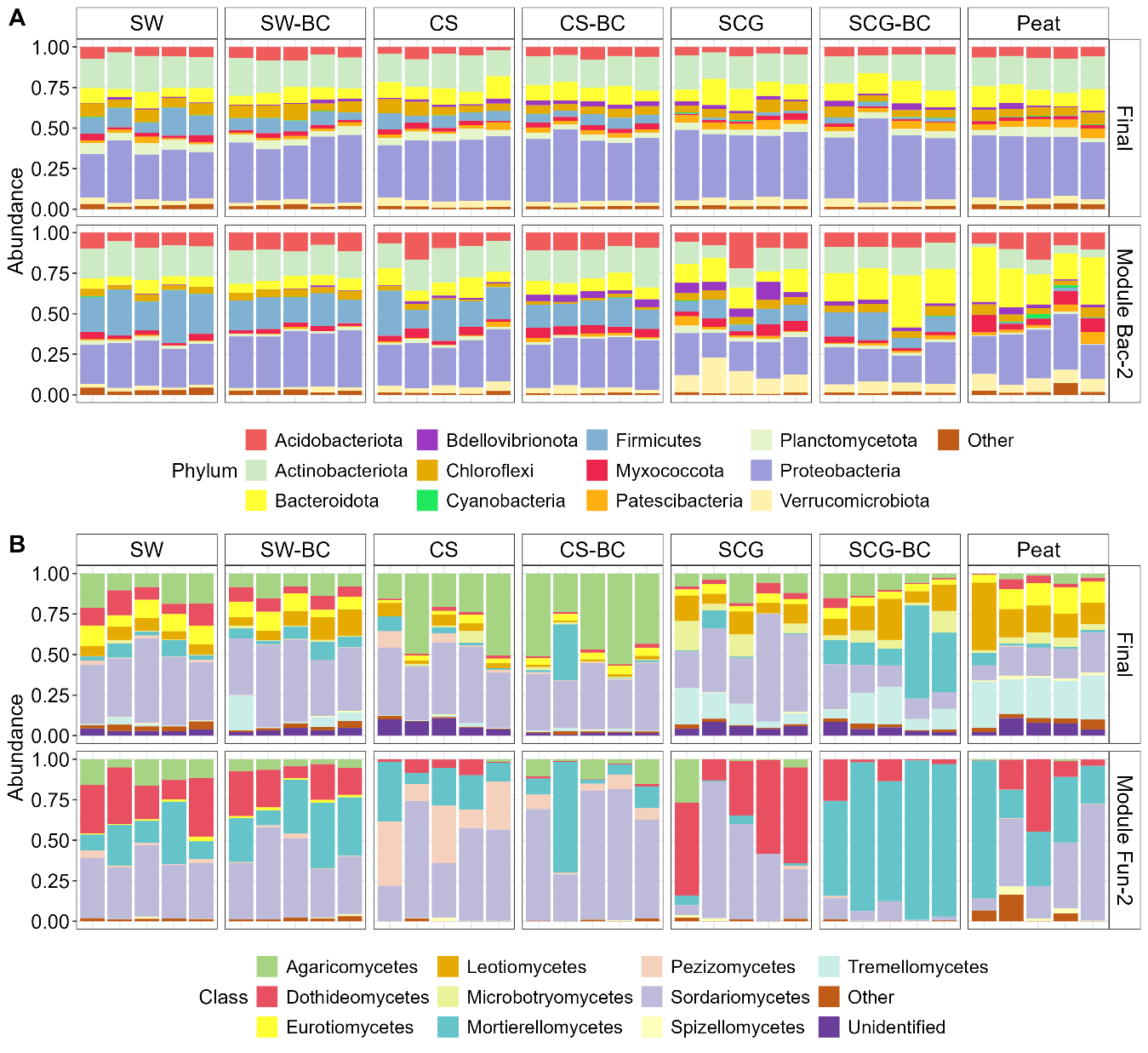


**Supplementary Figure S8.** Taxonomic composition of the complete prokaryotic and fungal communities and the subset belonging to Module-2 communities after tomato cultivation. **A)** Prokaryotic communities at the phylum level and **B)** fungi communities at the class level. “Other” includes minor prokaryotic phylum (relative abundance lower than 2.5%) and fungal class (lower than 5%), and “Unidentified taxonomically unassigned taxa. SW – Seaweed, CS – Coffee Silverskin, SCG – Spent Coffee Grounds, BC – Biochar was blended during composting.

**
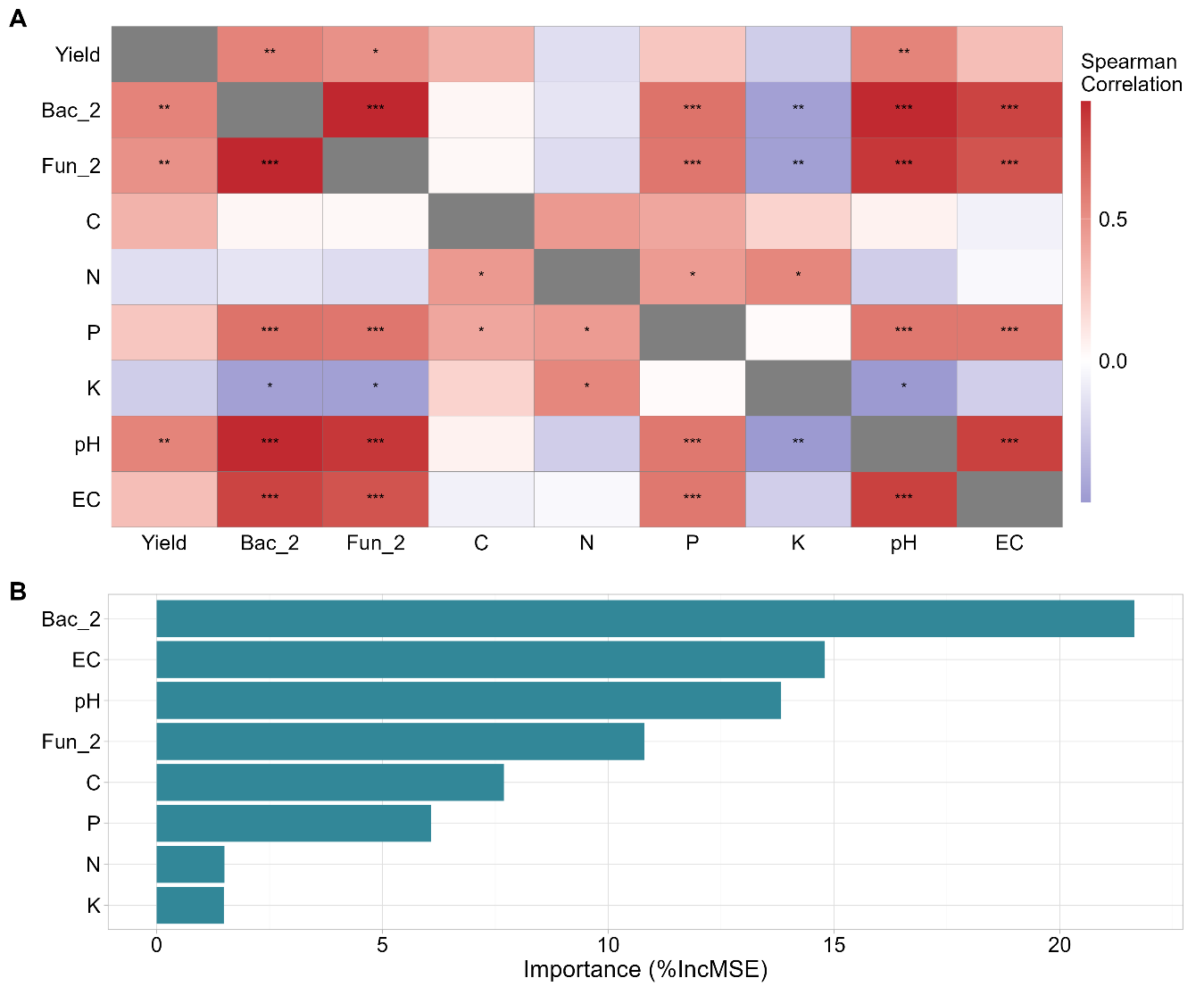
**

**Supplementary Figure S9.** Linear and non-linear relationships between tomato yield and substrate chemical and microbial properties. **A)** Spearman’s rank correlation. Asterisks denote the significance of each pairwise correlation (false discovery rate adjusted, **p* < 0.05, ***p* < 0.01, ****p* < 0.001). **B)** Random forest analysis to identify the relative importance of the different substrate chemical and microbial properties predicting tomato yield. The relative importance is estimated based on the increase in mean-square error (%IncMSE, R^2^ = 0.36 and MSE = 1.159).

**Supplementary Table S1:** PERMANOVA results comparing microbial community differences (bacteria, fungi) among feedstock material (seaweed, coffee silverskin or spent coffee grounds), between biochar application methods (blended during composting or at the bottom of the pot), and between sampling times (before or after harvest) (**Supplementary Figure S1**).

| **Community** | **Source of Variation** | **R^2^** | **F** | **Pr(>F)** |
| --- | --- | --- | --- | --- |
| Bacteria | Material | 0.356 | 15.189 | < 0.001 *** |
| Bacteria | Biochar application | 0.023 | 3.007 | 0.002 ** |
| Bacteria | Interaction | 0.043 | 2.747 | < 0.001 *** |
| Bacteria | Harvest | 0.100 | 12.864 | < 0.001 *** |
| Fungi | Material | 0.493 | 25.631 | < 0.001 *** |
| Fungi | Biochar application | 0.024 | 3.768 | 0.005 ** |
| Fungi | Interaction | 0.044 | 3.441 | < 0.001 *** |
| Fungi | Harvest | 0.040 | 6.307 | < 0.001 *** |

**Supplementary Table S2.** Standardized direct effects of all significant and non-significant paths considered in the structural equation model (SEM) from **Figure 4**.

| Response | Predictor | Standardized Estimate | P |
| --- | --- | --- | --- |
| Yield | Bacteria | 1.477 | 0.004 ** |
| Yield | Fungi | -0.140 | 0.674 |
| Yield | pH | 0.293 | 0.491 |
| Yield | EC | -0.819 | 0.004 ** |
| Yield | C | 0.130 | 0.470 |
| Yield | N | 0.139 | 0.598 |
| Yield | P | -0.468 | 0.153 |
| Yield | K | 0.142 | 0.383 |
| Bacteria | Material | - | 0.006 ** |
| Bacteria | Material = Peat | 0.291 ^×^ | < 0.001 *** |
| Bacteria | Material = SCG | 0.308 ^×^ | < 0.001 *** |
| Bacteria | Material = CS | 0.377 ^×^ | < 0.001 *** |
| Bacteria | Material = SW | 0.401 ^×^ | < 0.001 *** |
| Bacteria | Biochar | - | 0.011 * |
| Bacteria | Biochar = Pot | 0.324 ^×^ | < 0.001 *** |
| Bacteria | Biochar = Blended | 0.364 ^×^ | < 0.001 *** |
| Bacteria | pH | -0.014 | 0.927 |
| Bacteria | EC | 0.139 | 0.124 |
| Bacteria | C | -0.154 | 0.110 |
| Bacteria | N | 0.009 | 0.944 |
| Bacteria | P | 0.045 | 0.675 |
| Bacteria | K | -0.043 | 0.419 |
| Fungi | Material | - |  |
| Fungi | Material = Peat | 0.234 ^×^ | 0.130 |
| Fungi | Material = SCG | 0.205 ^×^ | < 0.001 *** |
| Fungi | Material = CS | 0.267 ^×^ | < 0.001 *** |
| Fungi | Material = SW | 0.342 ^×^ | < 0.001 *** |
| Fungi | Biochar | - | < 0.001 *** |
| Fungi | Biochar = Pot | 0.264 ^×^ | 0.921 |
| Fungi | Biochar = Blended | 0.261 ^×^ | < 0.001 *** |
| Fungi | pH | 0.116 | 0.722 |
| Fungi | EC | 0.083 | 0.649 |
| Fungi | C | 0.082 | 0.673 |
| Fungi | N | 0.139 | 0.501 |
| Fungi | P | 0.144 | 0.518 |
| Fungi | K | -0.126 | 0.242 |
| Bacteria | Fungi | 0.520 | 0.0011 ** |
| C | Material | - | < 0.001 *** |
| C | Material = Peat | 394.441 ^×^ | < 0.001 *** |
| C | Material = SCG | 404.579 ^×^ | < 0.001 *** |
| C | Material = CS | 467.782 ^×^ | < 0.001 *** |
| C | Material = SW | 346.511 ^×^ | < 0.001 *** |
| C | Biochar | - | < 0.001 *** |
| C | Biochar = Pot | 367.233 ^×^ | < 0.001 *** |
| C | Biochar = Blended | 439.423 ^×^ | < 0.001 *** |
| N | Material | - | < 0.001 *** |
| N | Material = Peat | 9.513 ^×^ | < 0.001 *** |
| N | Material = SCG | 13.552 ^×^ | < 0.001 *** |
| N | Material = CS | 11.943 ^×^ | < 0.001 *** |
| N | Material = SW | 6.924 ^×^ | < 0.001 *** |
| N | Biochar | - | < 0.001 *** |
| N | Biochar = Pot | 11.998 ^×^ | < 0.001 *** |
| N | Biochar = Blended | 8.968 ^×^ | < 0.001 *** |
| N | P | 0.638 | < 0.001 *** |
| P | Material | - | < 0.001 *** |
| P | Material = Peat | 0.332 ^×^ | < 0.001 *** |
| P | Material = SCG | 0.523 ^×^ | < 0.001 *** |
| P | Material = CS | 0.697 ^×^ | < 0.001 *** |
| P | Material = SW | 0.649 ^×^ | < 0.001 *** |
| P | Biochar | - | 0.284 |
| P | Biochar = Pot | 0.566 ^×^ | < 0.001 *** |
| P | Biochar = Blended | 0.534 ^×^ | < 0.001 *** |
| K | Material | - | < 0.001 *** |
| K | Material = Peat | 3.768 ^×^ | 0.002 ** |
| K | Material = SCG | 6.273 ^×^ | < 0.001 *** |
| K | Material = CS | 5.043 ^×^ | < 0.001 *** |
| K | Material = SW | 2.696 ^×^ | < 0.001 *** |
| K | Biochar | - | 0.199 |
| K | Biochar = Pot | 4.899 ^×^ | < 0.001 *** |
| K | Biochar = Blended | 3.991 ^×^ | < 0.001 *** |
| pH | Material | - | < 0.001 *** |
| pH | Material = Peat | 6.914 ^×^ | < 0.001 *** |
| pH | Material = SCG | 6.894 ^×^ | < 0.001 *** |
| pH | Material = CS | 7.258 ^×^ | < 0.001 *** |
| pH | Material = SW | 7.662 ^×^ | < 0.001 *** |
| pH | Biochar | - | 0.258 |
| pH | Biochar = Pot | 7.124 ^×^ | < 0.001 *** |
| pH | Biochar = Blended | 7.240 ^×^ | < 0.001 *** |
| pH | C | -0.013 | 0.918 |
| pH | N | -0.201 | 0.115 |
| pH | P | 0.350 | 0.007 ** |
| pH | K | -0.049 | 0.461 |
| EC | Material | - | 0.006 ** |
| EC | Material = Peat | 0.075 ^×^ | 0.133 |
| EC | Material = SCG | 0.090 ^×^ | 0.006 ** |
| EC | Material = CS | 0.181 ^×^ | < 0.001 *** |
| EC | Material = SW | 0.298 ^×^ | < 0.001 *** |
| EC | Biochar | - | 0.201 |
| EC | Biochar = Pot | 0.191 ^×^ | < 0.001 *** |
| EC | Biochar = Blended | 0.132 ^×^ | < 0.001 *** |
| EC | C | -0.262 | 0.156 |
| EC | N | -0.021 | 0.930 |
| EC | P | 0.240 | 0.264 |
| EC | K | 0.110 | 0.345 |

Asterisks denote the significance levels (****p* < 0.001, ***p* < 0.01, **p* < 0.05 and · *p* < 0.1).

^×^ Standardized estimates were not calculated for categorical variables.

**Supplementary Table S3.** Rationale behind each association link proposed for the structural equation modelling (SEM) in **Figure 4** and **Supplementary Figure S10**.

| **#** | **Associations** | **Rationale** | **References** |
| --- | --- | --- | --- |
| **1** | Material → Substrate properties, organic and nutrient concentrations | Organic substrates from different feedstock materials will have specific properties like texture, nutrient content or composition of organic matter. | (Xie *et al.* 2023) |
| **2** | Biochar → Substrate properties, organic and nutrient concentrations | Biochar improves substrate properties such as C and nutrient content, pH or water retention capacity. | (Gao *et al.* 2023; Sánchez-Monedero *et al.* 2019; Teodoro *et al.* 2020) |
| **3** | Substrate properties, organic and nutrient concentrations → Tomato Yield | Tomato yield is sensitive to substrate properties, especially those concerning plant nutrition and water content. | (Li *et al.* 2019; Maynard 1995; Yang *et al.* 2015) |
| **4** | Organic matter → Nutrients and other properties | The recycling of nutrients from organic matter is a crucial process to maintain fertility. In addition, the concentration of organic ions will alter pH and other properties such as electric conductivity. | (Ritchie & Dolling 1985; Tiessen *et al.* 1994) |
| **5** | Nutrient concentrations → Other properties | The concentration of certain nutrients has important impact on substrate pH and EC. | (Silber & Bar-Tal 2019) |
| **6** | Material → Microbial communities | The type of organic feedstock used during composting plays a crucial role in shaping microbial communities. | (Bender *et al.* 2016) |
| **7** | Biochar → Microbial communities | Biochar alter the pH of the mixtures, creates niches within its pore network and could retain nutrients due to entrapment or adsorption on its surface. | (Anderson *et al.* 2011; Palansooriya *et al.* 2019) |
| **8** | Substrate properties, organic and nutrient concentrations → Microbial communities | Substrate properties are key determinants of microbial communities. pH is among the strongest selective forces for microbes, together with water availability. | (Barnett *et al.* 2020; Hou *et al.* 2022; Liu *et al.* 2022; Yang *et al.* 2022) |
| **9** | Fungi ↔ Bacteria | Fungi and bacteria are found together in a wide variety of environments where they assemble into dynamic co-evolving communities. | (Deveau *et al.* 2018) |
| **10** | Microbial communities → Tomato Yield | Specific microbial communities have shown positive effects on crop productivity in tomato production. | (Anzalone *et al.* 2022; Sinno *et al.* 2020; Usero *et al.* 2021) |

**Supplementary Table S4**. Bulk densities (average ± standard deviation, n = 3) based on the dry mass of the raw materials, the peat-based horticultural substrate and the compost-based substrates calculated using the “tap density” method (Amidon *et al.* 2017).

| Materials | | Bulk Density (g/cm^3^) |
| --- | --- | --- |
| Feedstocks | Seaweeds | NA |
|  | Biochar | 0.24 ± 0.02 |
|  | SCG | 0.72 ± 0.02 |
|  | CSK | 0.50 ± 0.02 |
|  | GWP | 0.12 ± 0.01 |
|  | Peat | 0.24 ± 0.01 |
| Substrates | SCG-BC | 0.27 ± 0.01 |
|  | SCG | 0.26 ± 0.04 |
|  | CS-BC | 0.24 ± 0.01 |
|  | CS | 0.27 ± 0.01 |
|  | SW-BC | 0.32 ± 0.01 |
|  | BC | 0.34 ± 0.01 |

**References**

Amidon, G.E., Meyer, P.J. & Mudie, D.M. (2017). Particle, Powder, and Compact Characterization. In: *Developing Solid Oral Dosage Forms*. Elsevier, pp. 271–293.

Anderson, C.R., Condron, L.M., Clough, T.J., Fiers, M., Stewart, A., Hill, R.A., et al. (2011). Biochar induced soil microbial community change: Implications for biogeochemical cycling of carbon, nitrogen and phosphorus. *Pedobiologia*, 54, 309–320.

Anzalone, A., Mosca, A., Dimaria, G., Nicotra, D., Tessitori, M., Privitera, G.F., et al. (2022). Soil and Soilless Tomato Cultivation Promote Different Microbial Communities That Provide New Models for Future Crop Interventions. *Int. J. Mol. Sci*., 23, 8820.

Barnett, S.E., Youngblut, N.D. & Buckley, D.H. (2020). Soil characteristics and land-use drive bacterial community assembly patterns. *FEMS Microbiol. Ecol*., 96, fiz194.

Bender, S.F., Wagg, C. & Van Der Heijden, M.G.A. (2016). An Underground Revolution: Biodiversity and Soil Ecological Engineering for Agricultural Sustainability. *Trends Ecol. Evol*., 31, 440–452.

Deveau, A., Bonito, G., Uehling, J., Paoletti, M., Becker, M., Bindschedler, S., et al. (2018). Bacterial–fungal interactions: ecology, mechanisms and challenges. *FEMS Microbiol. Rev*., 42, 335–352.

Gao, S., Harrison, B.P., Thao, T., Gonzales, M.L., An, D., Ghezzehei, T.A., et al. (2023). Biochar co‐compost improves nitrogen retention and reduces carbon emissions in a winter wheat cropping system*. GCB Bioenergy*, 15, 462–477.

Hou, W., He, M., Qi, Y., Liu, T. & Luo, J. (2022). Soil nematode community assembly in a primary tropical lowland rainforest. *Front. Ecol. Evol*., 10, 1034829.

Li, J., Gao, Y., Zhang, X., Tian, P., Li, J. & Tian, Y. (2019). Comprehensive comparison of different saline water irrigation strategies for tomato production: Soil properties, plant growth, fruit yield and fruit quality. *Agric. Water Manag*., 213, 521–533.

Liu, S., He, F., Kuzyakov, Y., Xiao, H., Hoang, D.T.T., Pu, S., et al. (2022). Nutrients in the rhizosphere: A meta-analysis of content, availability, and influencing factors. *Sci. Total Environ*., 826, 153908.

Maynard, A.A. (1995). Cumulative Effect of Annual Additions of MSW Compost on the Yield of Field-Grown Tomatoes. *Compost Sci. Util*., 3, 47–54.

Palansooriya, K.N., Wong, J.T.F., Hashimoto, Y., Huang, L., Rinklebe, J., Chang, S.X., et al. (2019). Response of microbial communities to biochar-amended soils: a critical review. *Biochar*, 1, 3–22.

Ritchie, G. & Dolling, P. (1985). The role of organic matter in soil acidification. *Soil Res*., 23, 569.

Sanchez-Monedero, M.A., Cayuela, M.L., Roig, A., Jindo, K., Mondini, C. & Bolan, N. (2018). Role of biochar as an additive in organic waste composting. *Bioresour. Technol*., 247, 1155–1164.

Silber, A. & Bar-Tal, A. (2019). Nutrition of Substrate-Grown Plants. In: Soilless Culture. Elsevier, pp. 197–257.

Sinno, M., Ranesi, M., Gioia, L., d’Errico, G. & Woo, S.L. (2020). Endophytic Fungi of Tomato and Their Potential Applications for Crop Improvement. *Agriculture*, 10, 587.

Teodoro, M., Trakal, L., Gallagher, B.N., Šimek, P., Soudek, P., Pohořelý, M., et al. (2020). Application of co-composted biochar significantly improved plant-growth relevant physical/chemical properties of a metal contaminated soil. *Chemosphere*, 242, 125255.

Tiessen, H., Cuevas, E. & Chacon, P. (1994). The role of soil organic matter in sustaining soil fertility. *Nature*, 371, 783–785.

Usero, F.M., Armas, C., Morillo, J.A., Gallardo, M., Thompson, R.B. & Pugnaire, F.I. (2021). Effects of soil microbial communities associated to different soil fertilization practices on tomato growth in intensive greenhouse agriculture. *Appl. Soil Ecol*., 162, 103896.

Xie, S., Tran, H.-T., Pu, M. & Zhang, T. (2023). Transformation characteristics of organic matter and phosphorus in composting processes of agricultural organic waste: Research trends. Mater. *Sci. Energy Technol*., 6, 331–342.

Yang, L., Ning, D., Yang, Y., He, N., Li, X., Cornell, C.R., et al. (2022). Precipitation balances deterministic and stochastic processes of bacterial community assembly in grassland soils. *Soil Biol. Biochem*., 168, 108635.

Yang, L., Zhao, F., Chang, Q., Li, T. & Li, F. (2015). Effects of vermicomposts on tomato yield and quality and soil fertility in greenhouse under different soil water regimes. *Agric. Water Manag.,* 160, 98–105.
